# *GhLPF1* Associated Network Is Involved with Cotton Lint Percentage Regulation Revealed by the Integrative Analysis of Spatial Transcriptome

**DOI:** 10.1002/advs.202414175

**Published:** 2025-02-11

**Authors:** Hongyu Wu, Luyao Wang, Shengjun Zhao, Mengtao Gao, Junfeng Cao, Yupeng Hao, Li Yu, Ting Zhao, Siyuan Wang, Jin Han, Yumeng Zhu, Yongyan Zhao, Jie Li, Ke Nie, Kening Lu, Linyun Ding, Zhiyuan Zhang, Tianzhen Zhang, Xueying Guan

**Affiliations:** ^1^ Zhejiang Provincial Key Laboratory of Crop Genetic Resources Institute of Crop Science Plant Precision Breeding Academy College of Agriculture and Biotechnology Zhejiang University Hangzhou 300058 China; ^2^ Yazhou Bay Science and Technology City Hainan Institute of Zhejiang University Building 11, Yongyou Industrial Park, Yazhou District Sanya Hainan 572025 China; ^3^ National Key Laboratory of Crop Genetics & Germplasm Enhancement and Utilization Ministry of Agriculture Nanjing Agricultural University Nanjing 210095 China; ^4^ Key Laboratory of Plant Nutrition and Fertilization in Low‐Middle Reaches of the Yangtze River Ministry of Agriculture Nanjing Agricultural University Nanjing 210095 China; ^5^ School of Life Sciences Centre for Cell & Developmental Biology and State Key Laboratory of Agrobiotechnology The Chinese University of Hong Kong Shatin Hong Kong 999077 China; ^6^ Institute of Leisure Agriculture Jiangsu Academy of Agricultural Sciences Nanjing 210014 China; ^7^ Seed Production and Quality Control Research Center Hainan Seed Industry Laboratory Sanya Hainan China

**Keywords:** fiber regulation, population‐wide transcriptome analysis, post‐transcriptional regulation, spatial transcriptome

## Abstract

Cotton fibers, derived from the epidermis of the ovule, provide a sustainable natural fiber source for the textile industry. Traits related to fiber yield are predominantly determined by molecular regulations in the epidermis of the outer integument (OI) region of the cotton ovule. Here, we identify an R2R3 MYB transcription factor coding gene *GhLPF1* within the QTL‐LP‐ChrA06 locus for lint percentage (LP, percentage of lint to seed cotton) through constructing the 1‐Day Post Anthesis Cotton Ovule Spatial Transcriptome Atlas. *GhLPF1* is subjected as a downstream target of miR828 during fiber development. The direct downstream genes (DDGs) of GhLPF1 are biased to increased expression in *GhLPF1‐CR*, and are preferentially expressed in OI, so that GhLPF1 is primarily a transcriptional repressor to its DDGs. Population‐wide transcriptome analysis confirms that expression variation of GhLPF1‐DDGs is significantly biased to negative correlation with LP, among which a type I homeobox protein‐coding gene *GhHB6* is further validated to be the directly downstream gene of GhLPF1. Given these data, it is demonstrated that GhLPF1 mediates a regulation network in LP as a transcriptional repressor, which makes it a valuable functional marker for fiber‐trait improvement application from QTL‐LP‐ChrA06.

## Graphical abstract

Utilizing spatial transcriptome analysis, a novel R2R3 MYB coding gene *GhLPF1* is identified as a key regulator within the QTL‐LP‐ChrA06 interval. Phenotypic analysis of *GhLPF1* transgenic cotton plants further supports its crucial function in fiber development. Additionally, an integrative analysis of CUT&Tag‐Seq and RNA‐Seq data demonstrates that GhLPF1 acts as a transcriptional repressor for its directly downstream target genes.

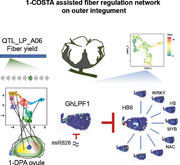

## 1 Introduction

Cotton fiber yield is a critical trait in cotton breeding. Over the past few decades, significant improvements have been made in fiber yield traits involved with lint percentage (LP) and seed index (SI) for cotton cultivars through selection.^[^ ^1^, ^2^ ^]^ However, the molecular regulation of causative genes in fiber yield traits remains largely unknown. Fiber development is controlled by a complex regulatory network governed by transcription factors (TFs), including R2R3 MYB, bHLH, WD, NAC, and homeodomain factors,^[^ ^3^, ^4^ ^]^ as reported lately. The R2R3 MYB TFs play crucial roles in fiber differentiation such as MML3, MML4, MYB2, and in fiber elongation such as MYB109, MYB7, MYBL1, and MYB5, ultimately impacting fiber yield and quality.^[^ ^5^, ^6^, ^7^, ^8^ ^]^

A group of R2R3 MYB TF coding genes are under conserved regulation by miR828 and miR858.^[^ ^9^ ^]^ Initially identified as a trigger for *TAS4*, generating small RNAs in a phased manner,^[^ ^10^ ^]^ miR828 targets a specific DNA sequence pattern, consistently associated with a dual‐hit site of miR858 at an interval of ≈12 nt.^[^ ^9^ ^]^ Research has shown that miR828 and miR858 primarily target a type of MYB TF gene involved in anthocyanin biosynthesis, a phenomenon reported in the genomes of potato,^[^ ^11^ ^]^ grape,^[^ ^12^, ^13^ ^]^ apple,^[^ ^9^ ^]^ kiwi fruit,^[^ ^9^, ^14^, ^15^ ^]^ and *Artemisia annua* L.^[^ ^16^ ^]^

The cotton gene *MYB2*, encoding an R2R3 MYB TF, was initially documented in 2014 for its function in complementing leaf trichome control in *Arabidopsis gl1*.^[^ ^17^ ^]^ Further studies reported that *GhMYB2* has cleavage sites for both miR828 and miR858.^[^ ^18^ ^]^ Upon recognition by miR828 and miR858, the *GhMYB2* mRNA can be processed into 21‐nt phasing RNAs.^[^ ^18^ ^]^ These phased small RNAs may act as feedback regulators during fiber development, to degrade the *GhMYB2* mRNA.^[^ ^19^ ^]^ While *GhMYB2* has been recognized as a significant regulator of plant trichome cells, its precise role in cotton fiber yield and quality traits remains unclear. The direct involvement of miR828 and its associated phased small RNAs in fiber development is not well‐defined. The debate surrounding the role of miRNA‐derived phasiRNA processing in crop traits persists due to a scarcity of comprehensive population‐wide data offering substantial evidence. The specific function of miR828/miR858 in cotton fiber agronomic traits still lacks elucidation.

MYB‐MIXTA‐like TFs, *GhMML3*/*GhMYB25‐like*,^[^ ^20^, ^21^ ^]^ and *GhMML4* ^[^ ^22^ ^]^ were reported to play a critical regulatory role in fuzz and lint cell differentiation, respectively. The function of *GhMML3* was validated by the single‐cell RNA‐Seq (scRNA‐seq) assays with early ovule outer integument (OI) tissue.^[^ ^23^, ^24^ ^]^ *GhMYB109*,^[^ ^25^ ^]^ *GhMYB7*,^[^ ^26^ ^]^ *GhMYBL1*,^[^ ^27^ ^]^ and *GhMYB212* ^[^ ^28^ ^]^ were also reported to impact fiber differentiation and elongation using reverse genetic approaches. *GhMYB5*, a candidate gene originating from a fiber length (FL) QTL, has been shown to increase FL with ectopic expression cotton lines.^[^ ^29^ ^]^ However, the downstream component of the cotton fiber‐related MYB TFs coding genes is barely reported, so far.

**Figure 1:**
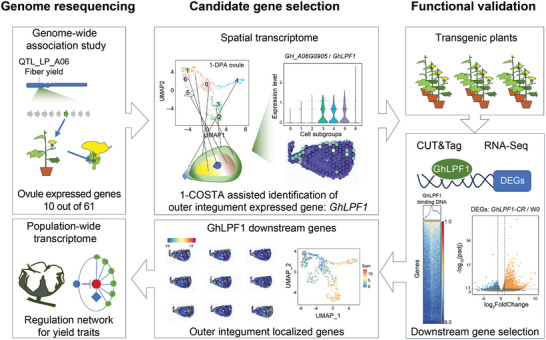
The workflow for this study to construct the fiber regulation network. The integrative analysis of the GWAS, QTL mapping, and spatial transcriptome in early cotton ovule was utilized to select the candidate gene *GhLPF1* for regulating cotton fiber yield. The *GhLPF1* transgenic cotton lines were developed to validate the function in fiber regulation. The integrative analysis of CUT&Tag‐Seq and RNA‐Seq revealed the downstream genes of *GhLPF1*, which were further validated by spatial transcriptome and additional population‐wide transcriptome analysis.

In this study, we constructed a workflow (**Figure** **1**) to characterize the functional regulation network associated with fiber yield. The QTL locus on chromosome A06 associated with LP was identified in the previous study through population genome resequencing and genome‐wide association study (GWAS) analysis.^[^ ^2^, ^30^ ^]^ Subsequently, the spatial transcriptome was used to assist in determining the effective gene *GhLPF1* in the QTL locus. The phenotypes of *GhLPF1* transgenic cotton further confirmed its function in regulating LP. Furthermore, RNA‐Seq and CUT and Tag‐Seq were integrated to find the direct downstream genes (DDGs) of GhLPF1, revealing that GhLPF1 is a transcriptional repressor to its DDGs. Through spatial transcriptome and population‐wide transcriptome analysis, we found that the DDGs of GhLPF1 were preferentially expressed in OI, and the expression variation of GhLPF1‐DDGs was significantly biased to negative correlation with LP. Our results provide a new R2R3 MYB coding gene *GhLPF1* and its associated network in modulating cotton fiber yield, which might facilitate future breeding to improve the fiber yield of upland cotton.

## 2 Results

### 2.1 *GhLPF1* is a Candidate Gene Associated with the QTL of LP on ChrA06

**Figure 2:**
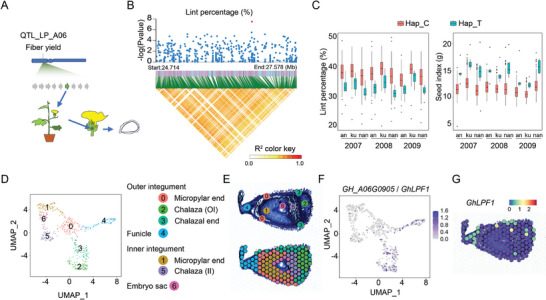
*GhLPF1* is a candidate gene associated with the fiber yield trait. A) The schematic chart shows the candidate gene selection in the fiber yield trait by narrowing down to the ovule‐specific expression genes. B) The Manhattan plot of GWAS results for LP on ChrA06. QTL_LP_ChrA06 is indicated by the red dot. The lower panel shows the pairwise linkage disequilibrium of this QTL. C) The box plots show that the haplotypes are significantly associated with the diverged traits of LP and SI in the upland cotton population. D) The UMAP analysis of the spatial transcriptome in 1‐DPA cotton ovule. Colors denote corresponding cell clusters. E) The gene atlas with classified cell types on the longitudinal section of 1‐DPA cotton ovule. F) The UMAP of *GH_A06G0905* / *GhLPF1* in the spatial transcriptome mapping. G) The spatial expression pattern of *GhLPF1* in 1‐DPA ovule.

We previously reported a stable QTL associated with LP on chromosome A06 (ChrA06) in upland cotton cultivars, named QTL‐LP‐ChrA06 (PVE = 12.88%; Effect = 4.08)^[^ ^2^, ^30^ ^]^ (**Figure** **2**A,B). Genomic variation on this QTL is correlated with LP and SI across multiple years and farming locations (*p* < 1E‐05) (Figure 2C). QTL‐LP‐ChrA06 (span from A06: 23 180 318 to 28 699 520) contains 61 protein‐coding genes (PCGs) in Upland cotton. Among them, ten genes showed expression in ovule and fiber tissues (RPKM > 1) (Figure S1, Supporting Information).

The lint fibers initiate from the ovule epidermis, beginning with the day post anthesis (0 DPA) to 3 DPA.^[^ ^31^ ^]^ 20–30% of ovule epidermal cells can differentiate into fiber cells, thereby the number of lint fiber initiation per ovule is an important factor in determining fiber yield.^[^ ^32^ ^]^ Bulk RNA‐seq cannot rule out the fine candidate genes in the epidermal cell layer of the ovule. To identify the major effective genes in this QTL‐LP‐ChrA06, a 1‐DPA cotton ovule spatial transcriptome atlas (1‐COSTA) was performed using 10× Genomics technology (Figure S2, Supporting Information). From each tissue section, a total of 531 spots were obtained, with a median of 4917 detected genes and 11004 UMI counts per spot (Figure S2, Table S1, Supporting Information). Utilizing uniform manifold approximation and projection (UMAP) analysis, the spots from the 1‐DPA cotton ovule spatial transcriptomics profiling were clustered into seven subgroups, representing the OI, funicle, inner integument (II) and embryo sac (ES) (Figure 2D, Table S2, Supporting Information). Notably, the fiber cell distributed on the ovule epidermis covered four distinct cell groups on 1‐COSTA: OI of the micropylar end, OI of the chalazal end, chalaza in OI, and part of the funicle (Figure 2D). Consequently, a gene atlas of cell types based on the region in 1‐DPA cotton ovule longitudinal sections, namely 1‐COSTA, was established and visualized (Figure 2D,E; Figure S3, Supporting Information).

Among the ten candidate PCGs in QTL‐LP‐ChrA06, *GH_A06G0905* and *GH_A06G0880* showed the active expression pattern specifically during fiber initiation stages from 0‐ to 3‐DPA and rapid elongation stages from 3‐ to 16‐DPA^[^ ^33^ ^]^ (Figure S1, Supporting Information). Furthermore, according to the UMAP and 1‐COSTA features, *GH_A06G0905* is expressed specifically in the epidermis of 1‐DPA ovules, indicating its potential role in fiber development (Figure 2F,G; Figure S4, Supporting Information). More specifically, *GH_A06G0905* is mainly expressed in three cell subgroups: chalaza in OI, OI of chalazal end, and funicle (Figure S5, Supporting Information). Therefore, *GH_A06G0905*, encoding a MYB transcription factor, was selected as a candidate gene associated with LP regulation in upland cotton, namely *GhLPF1* (LP factor 1). *GhLPF1* showed preferential expression in 1‐ and 3‐DPA ovules, with relatively low expression in the homeolog on the D sub‐genome (Figure S6, Supporting Information).

*GhLPF1* encodes a protein that shows 55% similarity to MYB66 and 43% to GL1 in *Arabidopsis* at the amino acid sequence level (Figure S7A, Supporting Information). The ectopic expression lines of *GhLPF1* in *Arabidopsis gl1* mutant with glabrous leaf and stem were generated to examine the function of *GhLPF1* in plant trichome regulation in general (Figure S8A, Supporting Information). The genomic and cDNA sequences of *GhLPF1* driven by the 35S promoter were transformed into the *gl1*. Both constructs were able to rescue the trichome development on the leaf and stem of the *gl1* mutant (Figure S8B–D, Supporting Information), which indicated that *GhLPF1* could activate the trichome development in plant epidermis.

### 2.2 The *GhLPF1* Regulates the Fiber Yield Traits in *Gossypium hirsutum*

**Figure 3:**
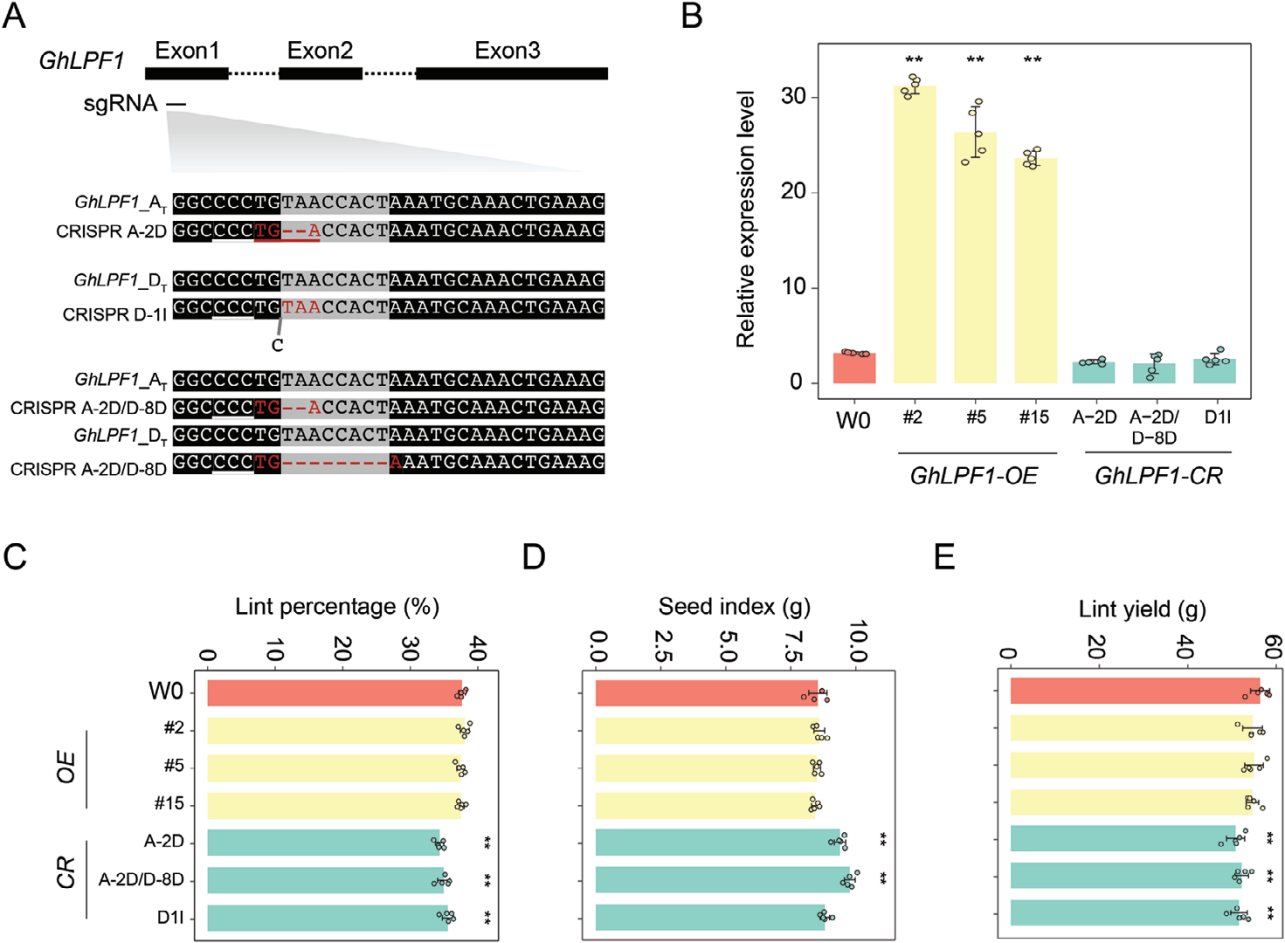
*GhLPF1* altered the LP and SI in the transgenic cotton. A) The schematic chart shows CRISPR‐editing sites of *GhLPF1* in *GhLPF1‐CR* lines. B) The histogram shows the relative expression level of *GhLPF1* in W0 and transgenic cotton lines. Values are means ± SD of five biological replicates (*n* = 5). The histograms show the LP (C), SI (D), and lint yield (E) of W0 and transgenic cotton lines. Values are means ± SD of five biological replicates (*n* = 5). The data were statistically analyzed by two‐tailed Student's *t*‐test (**p < 0.01).

To validate the function of *GhLPF1* in regulating cotton fiber, the ectopic expression lines (*35S::GhLPF1*, *GhLPF1‐OE*) and gene knockout lines (*GhLPF1‐CR*) were generated in the *Gossypium hirsutum* L. acc. W0 background (**Figure** **3**A). For each *GhLPF1‐CR* line, we designed one target targeting the conserved domain of both the *GhLPF1‐A_T_* (T indicates tetraploid) and its homeolog on the D subgenome (*GhLPF1‐D_T_*) (Figure 3A). Three homozygous lines of *GhLPF1‐CR* with the 2‐bp deletion on *GhLPF1‐A_T_* (A2D), 1‐bp insertion on *GhLPF1‐D_T_* (D1I), and double mutant with A2D and 8‐bp deletion on *GhLPF1‐D_T_* (A2D/D8D) were obtained, respectively (Figure 3A). The gene editing in both locations resulted in the early termination of the encoded protein (Figure 3A). Besides, multiple lines from the T_3_ generation of *GhLPF1‐OE* were obtained, showing the expected active transcription of *GhLPF1* (Figure 3B). The endogenous expression level of *GhLPF1* did not change significantly in the *GhLPF1‐CR* lines (Figure 3B). Taken together, three *GhLPF1‐OE* and three *GhLPF1‐CR* lines were used for further observation of fiber traits.

The whole plants of all transgenic lines did not show obvious phenotypic differences during plant growth (Figure S9, Supporting Information). The LP level was decreased in the *GhLPF1‐CR* lines (Figure 3C; Figure S10B, Supporting Information), which aligned with the increased SI (Figure 3D; Figure S10C, Supporting Information) and reduced lint yield (Figure 3E; Figure S10D, Supporting Information) for two farming seasons. However, the fiber cell initiation number observed in the 1‐DPA ovule epidermis did not show statistical differences between *GhLPF1‐CR* and W0 lines (Figures S10E and S11, Supporting Information). Surprisingly, the *GhLPF1‐OE* lines did not show significant differences in LP, and SI (Figure 3C,D; Figure S10B,C, Supporting Information). The phenotypic differences in the *GhLPF1‐CR* lines revealed that *GhLPF1* is a regulator in LP, which is consistent with its functional correlation in QTL_LP_ChrA06. Taken together, we suggested that the *GhLPF1* involved regulation is related to fiber development and seed size.

### 2.3 Regulation of *GhLPF1* at the Post‐Transcription Level under miR828

To elucidate the reason that *GhLPF1‐OE* lines did not show obvious phenotypic differences compared with W0, we first investigated the relationship between gene transcription activity and fiber traits. Correlation analysis was performed between *GhLPF1* mRNA levels and LP and SI in an upland cotton population. In multiple replicates from different farming years and locations, the variation of *GhLPF1* transcription showed a positive correlation with LP (*R* = 0.08–0.13, *p*‐value = 0.072–0.29) at 1‐DPA ovule (Figure S12, Supporting Information). However, the correlations were not up to statistical difference. The weak correlation between *GhLPF1* transcription variation and LP was also similar to the observations in the transgenic lines, which indicated an alternative genetic regulation mechanism of *GhLPF1* in fiber development.

**Figure 4:**
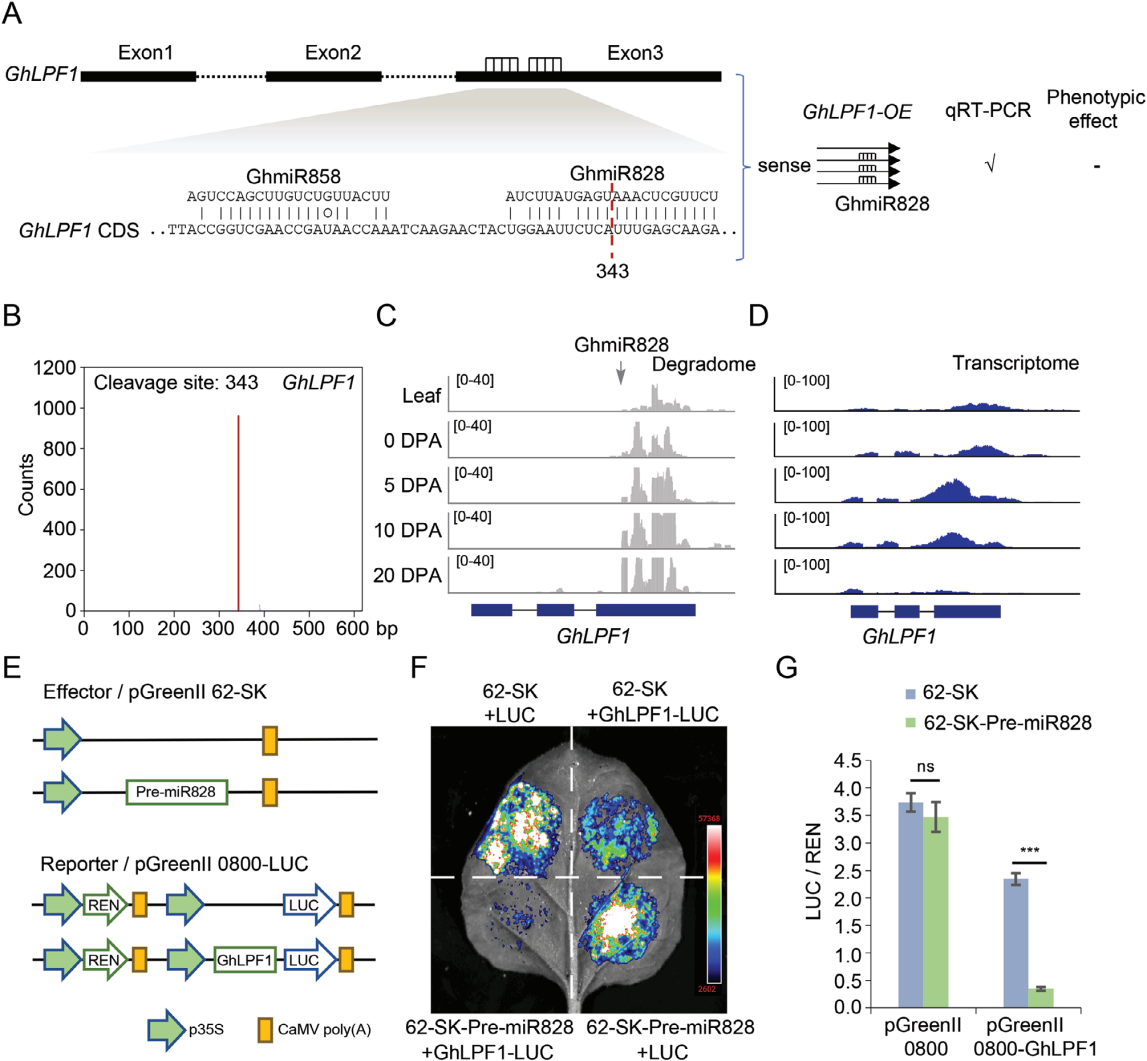
*GhLPF1* mRNA is regulated by miR828 at the post‐transcriptional level. A) The schematic chart shows that *GhLPF1* is targeted by miR828 and miR858 on Exon3. The hypothetical cleavage site of miR828 was predicated on 343‐bp downstream of ATG. B) The line chart shows the counts of the degraded transcript on the *GhLPF1* mRNA. C) The degradome data show the small RNA mapped onto the *GhLPF1*. The arrow indicates the miR828 targeting site on exon 3 of *GhLPF1*. The detected tissues include leaf, 0‐DPA ovule, and fibers from 5, 10, and 20 DPA. D) The stack plots show the reads mapped onto the *GhLPF1* using the transcriptome data of the tissues identical to panel C. E) The schematic diagram of effectors and reporters which was used in dual‐luciferase reporter assays. Firefly luciferase (LUC) and renilla luciferase (REN) activities were used as indicators. F) The luciferase activities of the tobacco leave co‐transfected with the different combinations of the effectors and reporters in panel E. G) The histogram shows the quantative value of the LUC / REN ratio in the tests of panel F. Values are means ± SD of five biological replicates (*n* = 5). The data were statistically analyzed by two‐tailed Student's *t*‐test (***p < 0.001, ns indicates no significant difference).

To reveal the presence of an additional layer of post‐transcriptional regulation on *GhLPF1*, small RNA targeting screening was performed, which showed that two reverse complementary sites for miRNAs were on the third exon of *GhLPF1*, with the potential to be degraded by miR858 and miR828 (**Figure** **4**A). Previous studies reported that cotton miR828 and miR858 were actively expressed in early ovules.^[^ ^18^ ^]^ Therefore, we hypothesized that the miRNA might cleave the endogenous *GhLPF1* transcripts to prevent the translation of the mRNA without interfering with the qRT‐PCR detection in *GhLPF1‐OE* lines (Figure 4A). To validate mRNA‐cleavage efficiency, degradome data^[^ ^34^ ^]^ from upland cotton fiber and leaf tissues were mapped against *GhLPF1*. A clear cleavage‐peak stack at the miR828 targeting site was observed in the degradome (Figure 4B). The degraded RNA fragments accumulated with the fiber elongation from 5‐ to 20‐DPA, reaching its peak during the mature stage, which was anti‐correlated with the *GhLPF1* RNA transcript stacking in the same developmental stages (Figure 4C,D). The data indicated that the miR828 cleavage on *GhLPF1* mRNA is associated with fiber development.

To confirm that miR828 can cleave *GhLPF1* mRNA, a miRNA digesting test was performed using the dual‐luciferase reporter assay. When co‐infection with the effector construct fused with Pre‐miR828 and reporter construct fused with *GhLPF1* cDNA, LUC/REN activity decreased by ≈90% compared to the combination of an empty effector and the same reporter construct, while LUC/REN activity of the empty reporter construct exhibited no obvious changes regardless of co‐infection with the empty effector or effector fused with Pre‐miR828 (Figure 4E–G). These results confirmed that endogenous *GhLPF1* mRNA is regulated by miR828 at the post‐transcriptional level. The miR828‐*GhLPF1* circuit may form a relatively stable feedback loop to buffer the transcription variation of *GhLPF1* during fiber development.

### 2.4 Genome‐Wide Identification of the Downstream Genes of *GhLPF1*

**Figure 5:**
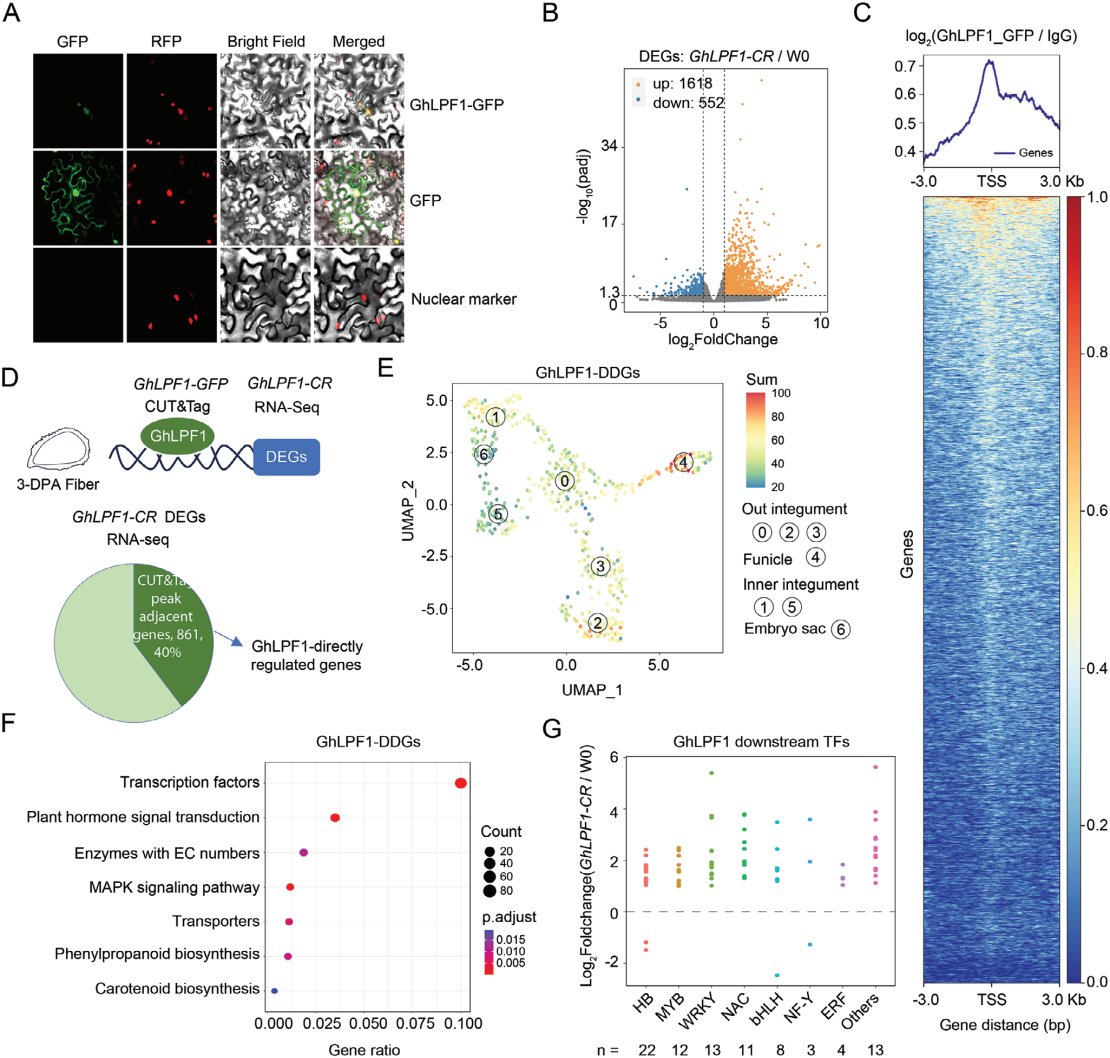
GhLPF1 is a transcriptional repressor in the ovule epidermal layer. A) The photo images show the subcellular location of GhLPF1:GFP fusion protein in tobacco leaf. B) The volcano plot shows the DEGs between the *GhLPF1‐CR* transgenic line and W0. C) The heatmap and signal density plots show the normalized GhLPF1:GFP/IgG binding peaks were distributed at the TSS of coding genes. D) The pie diagram shows that 40% of the DEGs between *GhLPF1‐CR* and W0 were adjacent to GhLPF1 binding peaks. E) The UMAP plot of GhLPF1‐DDGs distribution on 1‐COSTA. F) The KEGG enrichment of the GhLPF1‐DDGs. G) The dot plot shows that the GhLPF1 downstream TFs shown in panel F were biased to up‐regulated in *GhLPF1‐CR* compared with W0 (log_2_Fold Change (*GhLPF1‐CR* / W0) > 0).

The protein encoded by *GhLPF1* shows homology to *MYB66* and *GL1* in *Arabidopsis*, which are transcription factors involved in regulating trichome development. Sub‐cellular localization studies revealed that GhLPF1::GFP is present in the nuclei (**Figure** **5**A), suggesting that the protein encoded by *GhLPF1* is highly like a transcription factor. To characterize the molecular function of *GhLPF1* in fiber development, the 3‐DPA fibers of *GhLPF1‐CR* and W0 were harvested for transcriptome sequencing (Table S3, Supporting Information). The differentially expressed genes (DEGs) in the *GhLPF1‐CR* were biased to activation (1618 genes were upregulated and 552 genes were downregulated) (Figure 5B; Table S4, Supporting Information).

To further find the potential DNA fragments that GhLPF1 directly interacts with, CUT&Tag technology was performed employing the GFP antibody (Figure S13A, Supporting Information). The overexpression of *GhLPF1* in *GhLPF1‐OE* lines did not significantly impact fiber traits. Nevertheless, it is probable that GhLPF1::GFP in *GhLPF1‐OE* lines reflects the endogenous behavior of GhLPF1 as a transcription factor acting on its downstream gene. Therefore, the 3‐DPA fibers of the *GhLPF1*‐*OE* line were harvested for the CUT and Tag DNA library construction, with two technical replicates (Table S5, Figure S13B–D, Supporting Information). The width of most peaks was ≈300 and 800 bp (Figure S13D, Supporting Information). The 37137 common peaks were called from two replicates (Table S6, Figure S13E, Supporting Information). Genome‐wide distribution analysis revealed that the GhLPF1‐GFP binding sites were highly enriched near the transcriptional start site (Figure 5C). Approximately 45% of the peaks were distributed in the distal intergenic region, while 47% of peaks were distributed in the promoter region (Figure S13F, Supporting Information). Among the 37 137 common peaks, 17 688 peaks (47.6%) were located at 3‐kb upstream of the transcription start site (TSS) of the adjacent coding genes, which were used for subsequent analysis. Subsequently, the DNA fragments of 17 688 peaks were submitted to predict the conserved DNA motif with Homer. The top‐ranking motif showed high similarity with the previously reported MYB binding motif of 5′‐AACNGC‐3′,^[^ ^35^ ^]^ which indicated that GhLPF1 harbors the characteristic of the MYB gene family (Figure S13G, Supporting Information). Furthermore, according to the Gene Ontology (GO) analysis, the genes with the GhLPF1 binding peaks on promoter region were enriched in the function of DNA binding and transcription regulator activity (Figure S14A, Supporting Information). The KEGG enrichment analysis revealed that the GhLPF1 binding peaks were enriched with signaling transduction pathways (Figure S14B, Supporting Information). According to the tissue expression pattern analysis, these GhLPF1‐activated genes are preferentially expressed in the 0‐, 1‐ and 3‐DPA ovules, which are at the fiber initiation stage (Figure S14C, Supporting Information).

Among the DEGs between *GhLPF1‐CR* and W0, 40% (861 out of 2170 genes) had GhLPF1 directly binding peaks (Figure 5D; Table S7, Supporting Information). These 861 genes were assigned as GhLPF1 directly downstream genes (GhLPF1‐DDGs). The 1‐COSTA UMAP of GhLPF1‐DDGs was enriched in the OI and funicle tissues (Figure 5E). The 861 GhLPF1‐DDGs with GhLPF1 directly binding peaks were enriched with TF coding genes (Figure 5F, Table S8, Supporting Information). Among these TF coding genes, a dominant proportion of genes (93%, 82 out of 88 genes) were activated in the *GhLPF1‐CR* ovule (Figure 5G). The TFs included the categories of HB, MYB, WRKY, NAC, bHLH, and others, with 22 HB TFs at the top (Table S8, Supporting Information). The transcriptional effects led to the potential that GhLPF1 is a transcriptional repressor.

### 2.5 GhLPF1 is Involved with the LP Regulation Network as a Transcription Factor

**Figure 6:**
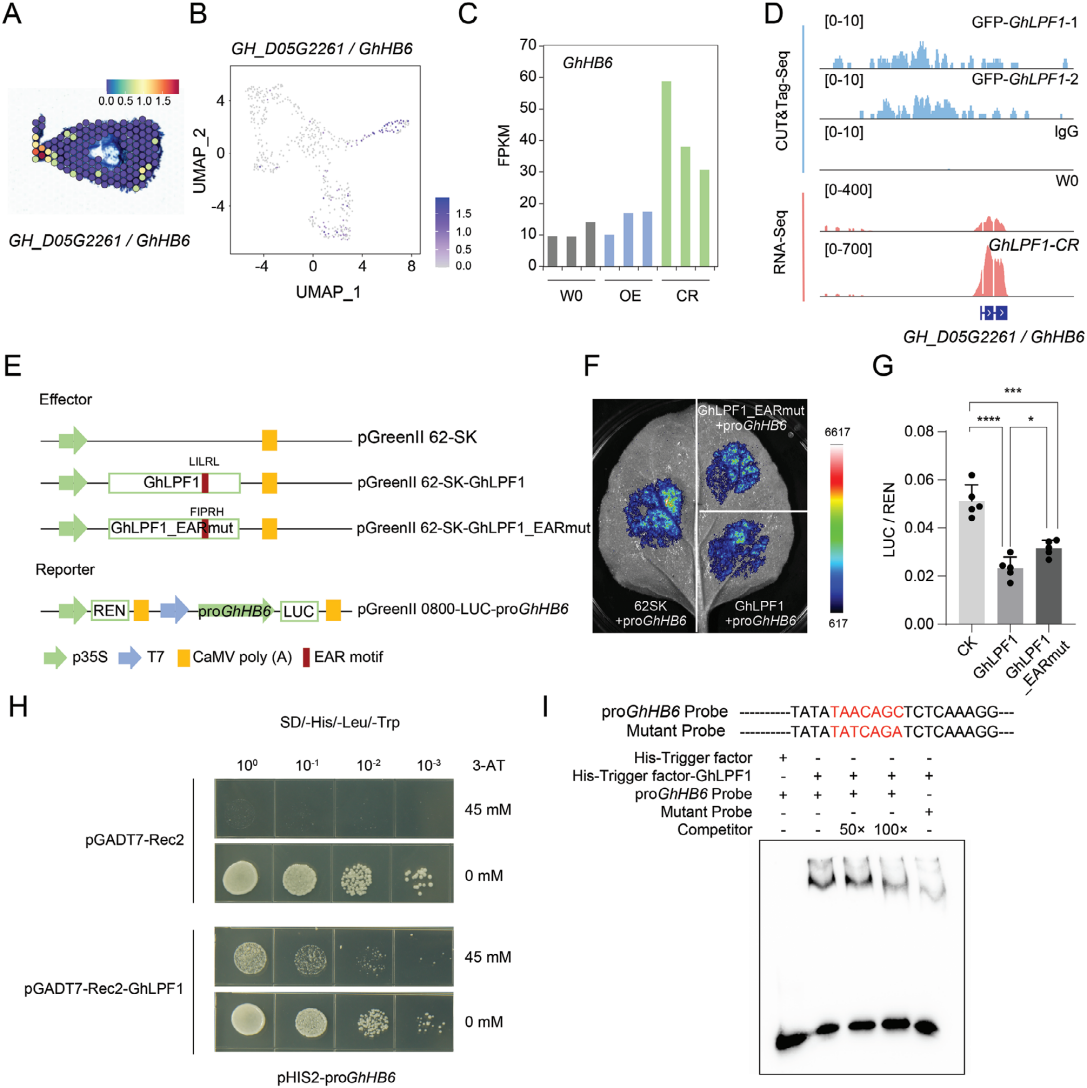
The GhLPF1 directly represses the expression of *GhHB6*. A) The spatial expression pattern of *GhHB6* in 1‐DPA ovule. B) The UMAP plot of *GhHB6* in 1‐COSTA. C) The histogram shows the expression level of *GhHB6* in cotton ovules of the W0 and *GhLPF1* transgenic cotton lines. D) The genome browser view of the *GhHB6* locus from the CUT&Tag‐Seq and RNA‐Seq data of GhLPF1, respectively. E) The schematic chart shows the dual‐luciferase reporter assay for GhLPF1 on the promoter of *GhHB6*. F) The image shows the luciferase activity using transient expression tobacco leaf. G) The histogram shows the quantitative value of the LUC / REN ratio using transient expression tobacco leaf in panel F. Values are means ± SD of five biological replicates (*n* = 5). Pair comparisons were made by a two‐tailed Student's *t*‐test (* indicates *P* < 0.05, *** indicates *P* < 0.001, **** indicates *P* < 0.0001). H) The yeast‐one hybrid assays show the binding capability of GhLPF1 on the promoter of *GhHB6*. I: EMSA assay shows that GhLPF1 could bind with the “AACAGC” motif on the promoter of *GhHB6*. The MYB binding motif on the promoter of *GhHB6* was mutated as shown in the mutant probe.

*GH_D05G2261*/*GhHB6*, encoding a type I homeobox (HD I) protein, was chosen from the activated TFs pool to validate whether it is a direct target of GhLPF1. *GhHB6* is predominantly expressed on the OI region of 1‐DPA ovule in 1‐COSTA mapping (**Figure** **6**A,B). The transcription activity of *GhHB6* in the *GhLPF1‐OE* did not differ from the transgenic background but was significantly activated in *GhLPF1‐CR* lines (Figure 6C). The promoter region of *GhHB6* showed a binding peak of GhLPF1 in CUT and Tag‐Seq (Figure 6D). To further confirm the binding capacity of GhLPF1 to the *GhHB6* promotor, the dual‐luciferase reporter assay was performed (Figure 6E). We found that GhLPF1 suppressed the activity of the *GhHB6* promotor compared with the control (CK) (Figure 6F,G). Moreover, the functional domain prediction identified an ethylene‐responsive element binding factor‐associated amphiphilic repression (EAR) motif on GhLPF1 (Figure 6E), which is reported as a conserved transcriptional repressor in *Arabidopsis*, rice, sorghum, and grapevine.^[^ ^36^ ^]^ The mutation of the EAR motif attenuated the suppression effect of GhLPF1 on the *GhHB6* promotor (Figure 6F,G). These results further confirmed that GhLPF1 is a transcriptional repressor for its regulated genes. The binding activity of GhLPF1 on the *GhHB6* promoter was further validated using yeast‐one hybrid (Y1H) (Figure 6H) and electrophoretic mobility shift assay (EMSA) (Figure 6I). Based on the tested assays and analyzed results, we suggested that GhLPF1 could directly bind with the *GhHB6* promoter and repress the expression of *GhHB6*. The natural variation of *GhHB6* in the upland cotton cultivated population is associated with LP (Figure S15, Supporting Information), which confirmed the function of *GhHB6* in cotton yield regulation.

**Figure 7:**
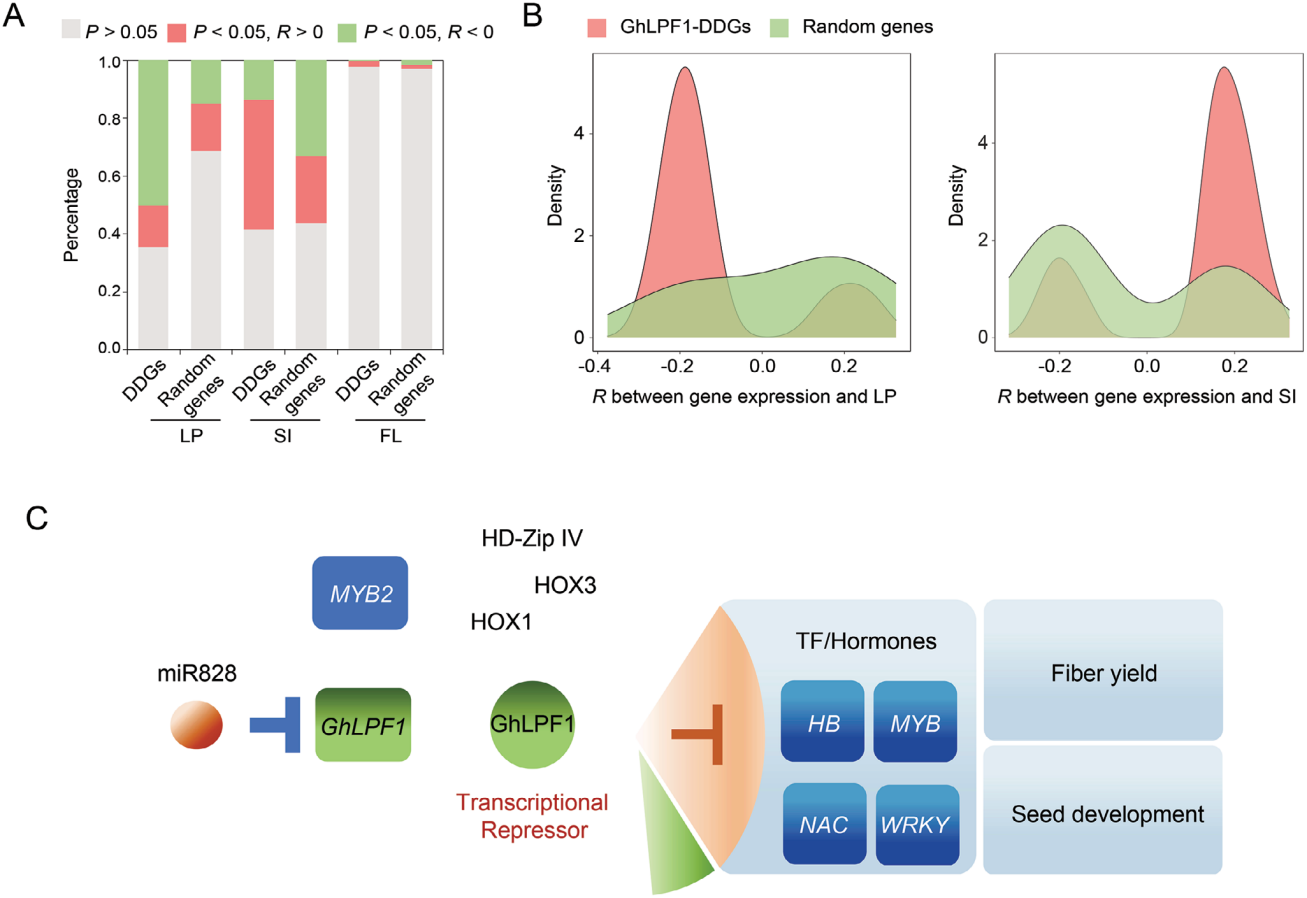
The *GhLPF1* is a functional marker for LP as a transcriptional repressor in the upland cotton population. A) The percentage of three different correlation relationships regarding the expression level of 861 GhLPF1‐DDGs and yield traits (LP and SI) in CUCP1. Pearson correlations and *P*‐value were calculated using the R command: cor and cor.test, respectively. The fiber quality trait of FL was used as a negative control for phenotype. 861 random genes were pulled out from the upland cotton genome to serve as a random control. The CUCP1 was adapted from the previous study^[^ ^2^, ^30^ ^]^ with 258 accessions in total. B) The density plots show the distribution of the correlation coefficient (*R*) of GhLPF1‐DDGs expression level and yield traits (LP and SI) in CUCP1. 861 random genes were pulled out from the upland cotton genome to serve as a random control. C) The working model for the molecular regulation network of *GhLPF1* for cotton fiber development.

The *GhLPF1‐CR* exhibited decreased LP and improved SI as shown in (Figure 3), which indicated that GhLPF1 is a positive regulator of LP. Therefore, if GhLPF1 was a transcriptional repressor, its downstream genes are hypothetically negative regulators to LP. To validate this hypothesis, the expression variation in China upland cotton population 1 (CUCP1, *n* = 258)^[^ ^2^, ^30^ ^]^ was examined for GhLPF1‐DDGs on the genetic impacts on agronomic traits. The expression of GhLPF1‐DDGs was significantly biased to the negative correlation group for LP in multiple farming years and locations (**Figure** **7**A,B; Figures S16 and S17, Supporting Information). The trend was anti‐correlated in SI (Figure 7A,B, Figures S18 and S19, Supporting Information). The FL was used to evaluate the GhLPF1‐DDGs function in power on other traits with nearly zero influence (Figure 7A; Figure S20, Supporting Information). These results indicated that GhLPF1 serves as a transcriptional repressor to downregulate LP negative regulators, which ultimately has a positive effect on LP.

## 3 Discussion

### 3.1 Construction of the Spatial Expression Atlas for Cotton Fiber and Seed Regulatory Genes through Spatial Transcriptome

Cotton fiber is a kind of single cell that differentiates from the epidermis of the ovule.^[^ ^37^ ^]^ The ovule epidermis constitutes the first layer of OI tissue, which provides the nutrients and cell developmental signals. Therefore, the molecular regulation components of fiber are theoretically assigned to the epidermal layer of the OI. Numerous studies have investigated the mechanisms of fiber development through constructing expression profiling in the epidermis of ovules and fibers. Since the early 21st century, highly expressed cDNAs were identified in cotton fibers or ovule epidermal cells using cDNA arrays,^[^ ^38^, ^39^ ^]^ after that, several expression profiles have been performed on various stages of fiber development,^[^ ^40^ ^]^ between different cotton species,^[^ ^41^ ^]^ between normal cotton and fiber‐related mutants.^[^ ^42^ ^]^ Multiple functional fiber genes, such as *MYB2*,^[^ ^17^ ^]^ *RDL1*,^[^ ^39^ ^]^ and *MML3* ^[^ ^23^ ^]^ were validated to express in fiber cells with RNA in situ technology. The promoters of *FBP7* and U6 were introduced in transgenic cotton to drive the expression of auxin biosynthesis gene *iaaM* specifically in the ovule epidermis to activate more fiber cell differentiation.^[^ ^43^ ^]^ What's more, suppression of sucrose synthase activity by at least 70% in the ovule epidermis,^[^ ^7^ ^]^ or of a vacuolar invertase gene *GhVIN1*,^[^ ^8^ ^]^ led to a fibreless phenotype. Therefore, the genes on the epidermis of the ovule take primary role in fiber cell fate.

For lint percentage, which is derived from the ratio of lint out of whole seed including fibers and seed, is also highly affected by seed size. Seed size is determined by both the enlargement of the integument, endosperm, and the primary cotyledon at the maturation stage. These indicate the genes under the epidermis may also play a role in lint percentage by altering seed size. In particular, the genes located in the cell layer beneath the epidermis of the outer integument are likely to play a significant role in both fiber cell fate differentiation and seed size determination.

So far, cotton fiber development is recognized as a unicellular progress with a unique spatial pattern. Fiber cell differentiation actively occurs in the epidermis of the ovule during early development. Laser capture microdissection (LCM) technology was applied to isolate the cotton ovule epidermal layer cells to construct bulk RNA transcriptome.^[^ ^39^ ^]^ Despite the higher sequencing depth of bulk RNA‐seq, LCM technology demands a prerequisite understanding and meticulous technical expertise,^[^ ^44^ ^]^ and it is difficult to separate the target tissue from other tissues completely. The latest studies employed scRNA‐seq technology to isolate the fiber cells from the OI of early cotton ovules.^[^ ^23^, ^24^ ^]^ Meanwhile, the spatial transcriptome was used to map transcripts to their corresponding tissue positions using unique barcodes to identify spatial gene expression patterns.^[^ ^44^ ^]^ Spatial transcriptome can provide a more intuitive display of mRNA distribution at high throughput and high accuracy of tissue specificity, which gives more confirmative evidence on gene function.

### 3.2 The Spatial Transcriptome Assists in Mining Candidate Genes within the QTL Interval and Verifying the Gene Regulatory Network

The major effective or causal gene navigation of QTLs has always been a major challenge in crop genetics and population genetics studies. Due to the continuous change in the genetic effects of quantitative traits, there are additive effects among multiple genes and loci, as well as the combined effects of numerous minor loci that contribute to the variation in quantitative traits. Various types of unknown variations can form the genetic basis of quantitative trait variations. In cotton, population‐wide study and QTL analysis are crucial to understanding the genetic basis for fiber yield. However, QTLs identified by GWAS or other population analyses typically cover large genomic regions, spreading over 2 Mb and encompassing tens of coding genes.^[^ ^2^, ^30^ ^]^ This presents a challenge in pinpointing the causal genes in the QTL locus without additional technical assistance.

Given the differentiation and initiation of fiber cells from the ovule epidermis, the spatial transcriptome can provide a gene expression atlas for fiber cells in the ovule longitudinal section. In this study, the integrated application with QTL mapping and 1‐COSTA demonstrated a comprehensive workflow to identify the fiber cell regulation network. 1‐COSTA provides clues for identifying the major effective gene *GhLPF1* in QTL‐LP‐ChrA06. According to 1‐COSTA, *GhLPF1* is expressed specifically in the epidermis of 1‐DPA ovules (Figure 2F,G). Moreover, the DDGs of GhLPF1 also exhibit preferential expression in the OI and funicle tissues (Figure 5E). The spatial expression patterns of GhLPF1 and its downstream genes reveal their potential roles in regulating fiber development. As a result, 1‐COSTA was proved to be an efficient map in assisting cotton fiber and seed developmental studies.

While scRNA‐Seq in eQTL mapping has been emerging as a promising research approach in animal populations and cell lines,^[^ ^45^, ^46^, ^47^, ^48^, ^49^, ^50^, ^51^ ^]^ to our knowledge, the application of spatial transcriptome in QTL mapping has not yet been reported in plants. The validation capability of 1‐COSTA shown in this study is at a high confidence level, with comparable potential applications for population‐wide crop studies as shown in animal studies. This study attempts to infer key genes within QTL intervals based on cell‐specific expression specificity, rooted in the precise cell types of cotton fiber development based on epidermal and dermal cells. This inference, supported by subsequent transgenic plant phenotypes, analysis of TF binding promoter sequences, population expression profiles of downstream regulatory network genes, and population genetic yield trait genotyping analyses, which demonstrates the significant role of *GhLPF1* itself in regulating cotton fiber yield.

### 3.3 The *GhLPF1*‐Mediated Regulation Network Forms a Negative Feedback Loop

*GhLPF1* encodes an R2R3 MYB TF within the locus of QTL‐LP‐ChrA06. Although the expression pattern of *GhLPF1* on cotton ovule OI provides strong evidence to support its function, its expression in the same population was not significantly affected by the genome structure variation. The overexpression of *GhLPF1* in the transgenic lines did not significantly affect the fiber trait. The function of miR828 on the *GhLPF1* transcript cleavage further revealed that the genome employs miRNA to prevent the transcription activity of *GhLPF1* itself at the post‐transcription level. Moreover, the RNA‐Seq and the promoter activity assay indicated that GhLPF1 is highly like a transcriptional repressor for downstream genes in 1‐DPA ovule. Based on the analyzed data, a molecular working model has been proposed (Figure 7C).

The Homeobox (HB) family comprises a group of homeodomain leucine zipper (HD‐Zip) transcription factors coding genes, which are involved in regulating plant epidermal trichome and fiber cell development. HD‐Zip includes type I, II, III, and IV subgroups.^[^ ^52^ ^]^ Compared with HD‐Zip III and IV, the HD‐Zip I subgroup lacks a START domain.^[^ ^52^, ^53^ ^]^ In *Arabidopsis*, GL2 is the first reported HD‐Zip IV type of TF as a positive regulator of leaf trichomes.^[^ ^54^ ^]^ Most of the reported HD protein in cotton fiber control is from the HD‐Zip IV subgroup. HOX1 is the first cotton HD‐Zip IV subgroup member reported as a functional homolog of GL2 in controlling plant leaf trichomes.^[^ ^55^ ^]^ HOX3 is one of the critical TF involved in cotton fiber elongation regulation.^[^ ^56^ ^]^ Besides, HOX3 forms a hub regulation network associated with HD1, DELLA, TCP4, PRE1, and miR319 to finely control the switch between fiber elongation and secondary cell wall assembly.^[^ ^34^, ^56^, ^57^ ^]^ Nevertheless, most of the GhLPF1 downstream HBs identified in this study belong to the HD‐Zip I subgroup. *GhHB6*, the downstream gene of GhLPF1, is the first reported HD‐Zip I TF involved with the regulation role of fiber development in cotton.

The leaf trichome repressors are reported as TRY, CPC in *Arabidopsis* trichome,^[^ ^58^, ^59^ ^]^ and as GL2, CPC, ETC1, and ETC2 in *Arabidopsis* root hairs.^[^ ^60^, ^61^ ^]^ TRY and CPC are MYB proteins as well but lack transcription activity domain.^[^ ^61^ ^]^ The widely accepted understanding of these negative regulators is that they act as competitors to full R2R3 MYBs, such as GL1, in binding with bHLH transcription factors (e.g., EGL3 and GL3).^[^ ^62^, ^63^ ^]^ It was reported that WDR in cotton fiber can directly bind with fuzz regulator MML3 and lint regulator MML4 without the involvement of any bridge protein in cotton.^[^ ^4^ ^]^ Therefore, it is hypothesized that the negative regulation in the fiber regulation complex is different in cotton compared to *Arabidopsis* leaf trichomes.

GhLPF1 has the complete functional structure as an R2R3 MYB TF, corresponding with its transcription function. The transcriptional repressor, EAR motif on the GhLPF1 transcription activity domain was confirmed as reported in *Arabidopsis*, rice, sorghum, and grapevine.^[^ ^36^, ^64^ ^]^ It was reported that, after fertilization, cotton ovules and fibers undergo a progression influenced by the phytohormones of auxin, ethylene, and so on.^[^ ^65^ ^]^ During the early ovule development, GhLPF1 activity could be associated with responses to ethylene via the EAR motif, likely mediating the crosstalk between multiple hormone‐signaling pathways.

### 3.4 The Future Perspective of *GhLPF1* in Cotton Breeding Applications

*GhLPF1* was identified in the co‐expression network of the fiber‐related gene *GhVIN1*,^[^ ^66^ ^]^ which indicates the important role of *GhLPF1* in fiber development. The natural variation in QTL‐LP‐ChrA06 links with *GhLPF1*, making it a valuable locus for fiber yield improvement. The LP is negatively correlated with seed size. In the *GhLPF1‐CR* lines, the LP was decreased, while the SI was improved. The GhLPF1‐DDGs were also associated with the seed yield variations. Meanwhile, *GhLPF1* encodes a TF with the capability of transcription regulation on other downstream TFs, which forms a potential regulation hub for LP and SI. Therefore, the application of *GhLPF1* in cotton fiber yield improvement requires more consideration of the impacts of SI variations.

We further examined the genomic structure‐associated expression variation in the 1‐DPA ovule profile. The expression variation of *GhLPF1* is weakly associated with LP. This natural variation in expression level provides an alternative clue to confirm the unchanged fiber trait in *GhLPF1‐OE* lines. We speculated that the manipulation of the miR828 targeting site may break down this buffering pattern to enhance the impact of *GhLPF1*. Cotton *MYB2* is one of the first fiber genes reported, also under the cleavage targeting of miR828.^[^ ^18^ ^]^ *GhLPF1* is a new MYB gene targeted by miR858 and miR828 in plants. The predominant expression activity of miR828 at an early ovule stage can effectively cleave its target transcripts, including *GhLPF1*, which creates a barrier to prevent the application of R2R3 MYB genes in fiber yield promotion. One strategy to avoid miR828 interference is to mutate the miR828 targeting site on the transcript.^[^ ^18^ ^]^

Alternatively, the downstream genes of *GhLPF1* were significantly associated with LP and SI in the upland cotton population. Specifically, *GhHB6* as an example shows that natural variations in upland cotton cultivars are associated with LP (Figure S15, Supporting Information). The majority of the cotton cultivars (906 out of 1031 accessions) have extensively utilized the elite allele of *GhHB6*, which is involved with the cotton yield regulation network mediated by *GhLPF1* in QTL_LP_ChrA06. The data reveal a major value for the application in cotton breeding for LP improvement.

## 4 Experimental Section

### Plant Material and Growth

The upland cotton (*Gossypium hirsutum*) genetic standard line, Texas Marker‐1 (TM‐1)^[^ ^67^ ^]^ obtained from the Agricultural Research Service, U.S. Department of Agriculture, and the Texas Agricultural Experiment Station, were cultivated in a growth chamber at 25 to 28 °C, with a light/dark cycle of 16/8 hours. The ovule tissues were harvested and frozen with liquid nitrogen for RNA extraction. *Arabidopsis thaliana* Col‐0 obtained from ABRC were cultivated in a growth chamber at 22 °C with a light/dark cycle of 16/8 hours.

### Transgenic Cotton and *Arabidopsis*

The constructs *GhLPF1‐OE* and *GhLPF1‐CR* were introduced into *Gossypium hirsutum* cv. W0 using *Agrobacterium tumefaciens*‐mediated transformation, as previously detailed.^[^ ^68^, ^69^ ^]^ The *GhLPF1‐OE* was transformed into *Arabidopsis* plants using the floral dip method.^[^ ^70^ ^]^ The homozygosity of transgenic plants was established by analyzing the kanamycin selection marker with PCR‐based genotyping. The primers utilized for vector construction and genotyping analysis are listed in Table S9 (Supporting Information).

### Constructs and Vectors

The total RNA was isolated from the 1‐DPA ovule by employing the SteadyPure Universal RNA Extraction Kit (AG21017, Accurate Biotechnology Co. Ltd) as per the manufacturer's instructions, including treatment with DNase I (Promega). Subsequently, the first‐strand cDNA was synthesized using M‐MLV reverse transcriptase (Promega). The open reading frames of *GhLPF1* were then amplified via regular PCR with *Xba*I and *BamH*I sites added, and subsequently inserted into the basic vector *pBI121* under the control of the constitutive Cauliflower mosaic virus 35S promoter. The primers used for vector construction and PCR‐based screening are provided in Table S9 (Supporting Information).

### Real‐Time Quantitative RT‐PCR (qRT‐PCR)

The qRT‐PCR was performed following the method described by Zhang et al.^[^ ^71^ ^]^ The primers used to detect the expression of *GhLPF1* are listed in Table S9 (Supporting Information). The internal control was *Histone3* from cotton. Three biological replicates were used for each reaction with two technical replicates each. Mean values and standard errors were calculated according to the data from three replicates.

### Dual‐Luciferase Reporter Assay

The full‐length coding sequence (CDS) of *GhLPF1* was cloned and inserted into the *pGreenII 0800–35S‐LUC* vector to obtain the reporter: *pGreenII 0800–35S‐GhLPF1‐LUC*.^[^ ^72^ ^]^ Simultaneously, the full length of *Pre‐miR828* was cloned and inserted into the *pGreenII 62‐SK* vector as an effector. Additionally, the full‐length CDS of *GhLPF1* was cloned and inserted into the *pGreenII 62‐SK* vector to obtain the effector: *pGreenII 62‐SK‐GhLPF1*. To get the mutated EAR motif of *GhLPF1*, the cloned full‐length CDS of *GhLPF1* served as the template, primers containing mutation sequences were designed to segmented amplify *GhLPF1* at the location of EAR motif, then ligated the fragments into an intact one by overlapping PCR. Similarly, the mutated *GhLPF1* was inserted into the *pGreenII 62‐SK* vector to obtain the effector: *pGreenII 62‐SK‐GhLPF1_*EARmut. Moreover, the promoter of *GhHB6* was cloned and inserted into the *pGreenII 0800‐LUC* vector as a reporter. The primers for the promoter and CDS cloning are listed in Table S9 (Supporting Information).

The indicated reporter and effector and their corresponding empty vectors were transformed into *Agrobacterium* strain GV3101 containing the *pSoup‐p19* helper plasmid. Various combinations of effector and reporter were co‐infiltrated into the 4‐week‐old tobacco leaves. After incubation at 22 °C (16‐h light/8‐h dark) for two days, the LUC fluorescence signal was detected by the chemiluminescence imaging system (Tanon‐5200), and the activities of firefly luciferase (LUC) and renilla luciferase (REN) were detected by the Dual‐Luciferase Reporter Gene Assay Kit (Yeasen, Cat. #11402ES60). Five biological replicates were carried out for the detection of luminescence intensity.

The subcellular location of GhLPF1 in tobacco leaves was determined as previously described.^[^ ^71^, ^73^ ^]^ GFP‐tagged proteins were subcloned in pBINGFP4 vectors for the transformation of tobacco leaves, respectively. The primers used are listed in Table S9 (Supporting Information). The RFP gene was used as the cytosol‐localized marker. Confocal analysis was performed on a Zeiss LSM780 confocal microscope using a 488 nm excitation laser for GFP and a 561 nm laser for RFP.

### Histochemical Staining

Histochemical localization of GUS was performed according to the method described by Jefferson.^[^ ^74^ ^]^ Fresh tissue was detached from the tobacco leaf and immediately exposed to X‐Gluc solution. After overnight incubation at 37 °C, stained samples were rinsed in 50%, 70%, and 100% ethanol for 5 min each and then bleached with 100% ethanol. Subsequently, the specimen was observed under a stereomicroscope (DP72, Olympus, Tokyo, Japan).

### Scanning Electronic Microscope

1‐DPA bolls were collected from transgenic cotton, and 3–4 ovules were taken from each boll in the same position and placed in a 2.5% glutaraldehyde aqueous solution for fixation. Scanning electronic microscopy (SEM) was performed using a modified protocol.^[^ ^39^, ^75^ ^]^ The samples were scanned and analyzed using a GeminiSEM 300 with an accelerating voltage of 3 kV and a working distance of 7 mm. Images were scanned and stored as TIFF files.

### 1‐DPA Cotton Ovule Spatial Transcriptome Atlas Construction/1‐COSTA

The protocol is modified from the previous publications.^[^ ^76^, ^77^ ^]^

### Plant Materials and Sampling

The upland cotton cultivar *Gossypium hirsutum* (accession TM‐1) was grown in the experimental greenhouse at Zhejiang University, Hangzhou, China. 1‐DPA ovules were collected in the afternoon (4:00 pm) and obtained by cutting using a surgical blade. The specimen was immersed into OCT (Sakura) compound within a mold, and placed in a vacuum on ice for 10 min. The sample was sliced at −20 °C cryochamber at a thickness of 10 µm.

### Tissue Optimization (TO)

Cryosections were performed on a Leica CM3050 and brightfield images were captured on a Leica Aperio Versa8 whole‐slide scanner at 20× resolution. The Visium Spatial Tissue Optimization Slide & Reagent kit (10× Genomics) was used to optimize permeabilization conditions for the tissue according to the Visium Spatial Tissue Optimization User Guide (CG000238, 10× Genomics). Briefly, the Visium Spatial Tissue Optimization workflow includes placing tissue sections on 7 Capture Areas on a Visium Tissue Optimization slide. The sections were fixed, stained, and then permeabilized at different times. mRNA released during permeabilization binds to oligonucleotides in the Capture Areas. Fluorescent cDNA was synthesized on the slide and imaged. The permeabilization time that results in the maximum fluorescence signal with the lowest signal diffusion was optimal. If the signal was the same at two‐time points, the longer permeabilization time was considered optimal.

### Visium Library Construction and NGS

The Visium Spatial Gene Expression Slide and Reagent kit (10X Genomics) was utilized to construct sequencing libraries following the guidelines outlined in the Visium Spatial Gene Expression User Guide (CG000239, 10× Genomics). A 10‐µm frozen tissue section was placed on one of the Visium gene expression slide capture areas in a slide. After tissue Toluidine Blue staining, bright‐field images were acquired as described in the Spatial Transcriptomics procedure. Tissue permeabilization was performed for an optimal minute, as established in the TO procedure. Then reverse transcription experiment was conducted and sequencing libraries were prepared following the manufacturer's protocol. Sequencing was performed by Novogene Co, Ltd., Beijing, China. Utilizing an Illumina Nova seq 6000 (Illumina, San Diego, CA) platform according to the manufacturer's instructions at an average depth of 300 million read‐pairs per sample.

### Processing of Spatial Transcriptome Sequencing Data

After trimming adapters and low‐quality bases, the clean data was loaded onto spaceranger‐2.0.0 count preprocessing. Software spaceranger mkref was employed to create a reference of *Gossypium hirsutum* TM‐1 v2.1. The spaceranger count program was used to get the spot‐gene matrix for downstream analyses. Then, Seurat‐4.3.0 (Hao et al. 2021) was employed to analyze the spatially‐resolved RNA‐seq matrix for dimension reduction, clustering, and visualization.

### CUT&Tag Assay

The CUT and Tag assay was performed as described^[^ ^78^ ^]^ with modifications. Ovules at 3‐DPA were collected from approximately five bolls of the *GhLPF1*‐OE transgenic line. Fibers isolated from the ovules were crosslinked using 1% formaldehyde for 20 min and stopped with 0.125 m glycine under vacuum. The crosslinked fibers were washed using ddH_2_O for three times. After washing, the fibers were transferred to paper towels to absorb excess water. The crosslinked fibers were then ground into powder in liquid nitrogen and suspended in buffer A (10 mm Tris pH 8.0, 10 mm KCl, 0.5 mm spermidine, 1 mm EDTA, β‐Mercaptoethanol). After filtration and centrifugation, the pellet was washed three times with buffer B (buffer A with 0.5% Triton X‐100 and 0.1% protease inhibitor cocktail (Calbiochem, Cat. #539133‐1SET).

The extracted nuclei were washed three times with wash buffer (10 mm Tris pH 8.0, 150 mm NaCl, 0.5 mm spermidine, 0.1% protease inhibitor cocktail). Then, the nuclei were incubated with primary antibody: anti‐GFP (Abcam, Cat. #ab290, 1:100 dilution), normal rabbit IgG (Millipore, Cat. #12‐370, 1:100 dilution) on a rotator at 4 °C overnight. The anti‐GFP antibody was used to bind with the GhLPF1‐GFP protein, and normal rabbit IgG served as a negative control. Two technical replicates were carried out for each antibody. The next day, the nuclei were incubated with a secondary antibody: guinea pig anti‐rabbit (Novus Biologicals, Cat. #NBP1‐72763, 1:100 dilution) for 1 h at room temperature on a rotator. Then the antibody‐binding nuclei were incubated with Hyperactive pA‐Tn5 Transposase (Vazyme, Cat. #S603‐01) for 1 hour on a rotator at room temperature.

For tagmentation, tagmentation buffer was added (10 mm Tris pH 8.0, 300 mm NaCl, 0.5 mm spermidine, 0.1% protease inhibitor cocktail, 10 mm MgCl2, 0.05% w/v digitonin) and incubated at 37 °C for one hour. A solution of 10% SDS and 0.5 m EDTA was added to stop the tagmentation reaction. After tagmentation, reverse crosslinking was performed at 65 °C overnight, and proteinase K was used to digest protein. DNA extraction and library construction were performed as described.^[^ ^78^ ^]^

### CUT and Tag Sequencing and Bioinformatic Analysis

Purified DNA libraries were sequenced on the Illumina platform to generate 150‐bp, paired‐end reads and obtained 4‐ to 5‐G clean base data for each library. Clean reads were aligned to the TM‐1 v2.1 reference genome using Hisat2 (v2.1.0) with parameters:

–no‐spliced‐alignment–no‐mixed–no‐discordant–phred33 ‐I 10 ‐X 700.^[^ ^79^ ^]^ PCR duplicates were removed using Picard Tools (v2.23.0) with MarkDuplicates. Enriched peaks were identified against IgG peaks using macs2 (v 2.1.3.3), with parameters: ‐g 2.29e10 ‐f BAMPE –keep‐dup all.^[^ ^80^ ^]^ Annotation of enriched peaks was performed using a R/Bioconductor package: ChIPseeker (v1.38.0).^[^ ^81^ ^]^ Peaks at the regions between −2 kb ahead of the TSSs and +2 kb after TSSs were selected as GhLPF1‐bound targets. The GO enrichment analyses were performed using the database g: Profile (http://biit.cs.ut.ee/gprofiler/)^[^ ^82^ ^]^ and agriGO (http://bioinfo.cau.edu.cn/agriGO).^[^ ^83^ ^]^ The KEGG enrichment analyses were performed using the database KEGG (https://www.kegg.jp/).^[^ ^84^ ^]^

### RNA‐Seq and Data Analysis

Ovules at 3‐DPA were harvested from *GhLPF1‐OE*, *GhLPF1‐CR* lines, and W0, with three replicates in each line. Subsequently, fibers were isolated from the ovule epidermis for RNA extraction. Prepared total RNA was sent to Novogene for library generation and sequencing (MGISEQ‐T7, PE150). Low‐quality bases were trimmed by TRIMMOMATIC.^[^ ^85^ ^]^ Clean reads were aligned to the TM‐1 v2.1 reference genome using Hisat2 (v2.1.0) with parameter (–dta).^[^ ^79^ ^]^ The reads mapped to the annotated TM‐1 transcripts^[^ ^86^ ^]^ were counted with the feature Counts algorithm (v2.0.1).^[^ ^87^ ^]^ The DEGs were identified with a filter (log_2_ FoldChange > 1 or < −1, FDR < 0.05) with the R (v4.2.3) package DESeq2 (v4.2.2).^[^ ^88^ ^]^

### Yeast One‐Hybrid Assay

The promoter region (2.0‐kb upstream of the ATG codon) of *GhHB6* was inserted into the pHis2 yeast vector (Clontech) as a bait‐reporter, and the full‐length CDS of *GhLPF1* was cloned and inserted into the pGADT‐Rec2 vector (Clontech) to produce prey protein. The pHis2‐pro*GhHB6* and pGADT‐Rec2‐*GhLPF1* constructs were co‐transformed into the Y187 strain, the pHis2‐pro*GhHB6*, and pGADT‐Rec2 were co‐transformed either to serve as a negative control. The protein‐DNA interaction was determined based on the growth of the co‐transformants on SD/‐His/‐Leu/‐Trp medium containing 45 mm 3‐amino‐1,2,4‐triazole (3‐AT, Coolaber, Cat. #CA1311‐25g). The primers for the promoter and CDS cloning are listed in Table S9 (Supporting Information).

### Electrophoretic Mobility Shift Assay

For expression and purification of the His‐GhLPF1 recombinant protein, the full‐length CDS of *GhLPF1* was inserted into a pCold TF vector (Tarkara, Cat. #3365). The construct was transformed into *E. coli* BL21 (DE3) competent cells (Tsingke, Cat. #TSC‐E06). The expression of His‐GhLPF1 recombinant protein was induced by 0.2 mm Isopropyl beta‐D‐thiogalactopyranoside at 16 °C for 48 h. The protein was purified by using the His‐tag Protein Purification Kit (Beyotime, Cat. #P2226) according to the manufacturer's manual. Native and mutated probes were synthesized and labeled with biotin using the EMSA Probe Biotin Labeling Kit (Beyotime, Cat. #GS008). EMSA assays were carried out using a Chemiluminescent EMSA Kit (Beyotime, Cat. #GS009) according to the manufacturer's manual. For the competition reaction, 50× and 100× unlabeled cold probes were mixed with the labeled probes. The DNA‐protein complex was separated by 6% native polyacrylamide gel electrophoresis and the signal of biotin was detected by the chemiluminescence imaging system (Tanon‐5200). The primers and probes used in this assay are listed in Table S9 (Supporting Information).

### Western Blot

3 DPA ovules harvested from W0 and *GhLPF1*‐OE transgenic lines were ground into powder in liquid nitrogen and suspended in NP‐40 lysis buffer with 1 mm PMSF (Solarbio, Cat. #N8032). After homogenization and centrifugation, the supernatant was then mixed with 5× SDS‐PAGE Sample Loading Buffer (Beyotime, Cat. #P0015) and denaturized at 98 °C for 10 min. The denaturized proteins were separated by 10% SDS‐PAGE gel and transformed to PVDF membrane for antibody incubation and chemiluminescence. The following antibodies were used: Anti‐GFP (Abcam, Cat. #ab290, 1:5000), Goat Anti‐Rabbit IgG H&L (HRP) (Abcam, Cat. #ab6721, 1:5000). The signal was detected by the chemiluminescence imaging system (Tanon‐5200).

### Statistical Analysis

The pre‐processing of data procedure for CUT and Tag‐Seq, RNA‐Seq, and spatial transcriptome were described in each method section. The sample size and statistical methods shown in each figure in the study were denoted in the legends. The students' *t*‐tests were used to evaluate the statistical significance between the two groups in the bar graphs. Values were presented as means ± SD. The significance was denoted as (*) *P*‐value < 0.05, (**) *P*‐value < 0.01, (***) *P*‐value < 0.001, and (****) *P*‐value < 0.0001, ns indicates no significant difference. GraphPad Prism8.0 was used to calculate the level of significance.

## Conflict of Interest

The authors declare no conflict of interest.

## Author Contributions

H.W., L.W., and S.Z. contributed equally to this work and co‐first authors. X.G., L.W., H.W., and T.Z. conceptualized the project. L.W. and T.G. created transgenic cotton and *Arabidopsis*. L.W., H.W., L.Y., J.H., Y.Z., Y.Z., J.L., K.L., K.N., and Z.Z. carried out the experiments. S.Z. performed 1‐COSTA. L.W., J.C., Y.H., S.W., and T.Z. performed the bioinformatics analysis. H.W., L.W., T.Z., and X.G. prepared the manuscript. All the authors read and approved the final manuscript.

## Supporting information



Supporting Information

Supporting Information

## Data Availability

The data that support the findings of this study are available in the supplementary material of this article.
