# Supplementary material for: GhLPF1 Associated Network Is Involved with Cotton Lint Percentage Regulation Revealed by the Integrative Analysis of Spatial Transcriptome
Source: Adv Sci (Weinh). 2025 Feb 11;12(13):2414175. doi: 10.1002/advs.202414175 (PMC11967919; doi:10.1002/advs.202414175)
Supplement: Supplementary file 1 — Supporting Information [file ADVS-12-2414175-s002.docx]

***GhLPF1* associated network is involved with cotton lint percentage regulation revealed by the integrative analysis of spatial transcriptome**

Hongyu Wu^1, #^, Luyao Wang^2, #^, Shengjun Zhao^1, 2, #^, Mengtao Gao^3, 4^, Junfeng Cao^5^, Yupeng Hao^1^, Li Yu^1^, Ting Zhao^1, 2^, Siyuan Wang^1^, Jin Han^1^, Yumeng Zhu^1, 2^, Yongyan Zhao^1^, Jie Li^3^, Ke Nie^1, 2^, Kening Lu^3^, Linyun Ding^3,6^, Zhiyuan Zhang^2^, Tianzhen Zhang^1, 2^, Xueying Guan^1 ,2, *^

^1^ Zhejiang Provincial Key Laboratory of Crop Genetic Resources, Institute of Crop Science, Plant Precision Breeding Academy, College of Agriculture and Biotechnology, Zhejiang University, Hangzhou, 300058, China.

^2^ Hainan Institute of Zhejiang University, Building 11, Yongyou Industrial Park, Yazhou Bay Science and Technology City, Yazhou District, Sanya, Hainan, 572025, China.

^3^ National Key Laboratory of Crop Genetics & Germplasm Enhancement and Utilization, Ministry of Agriculture, Nanjing Agricultural University, Nanjing, 210095, China.

^4^ Key Laboratory of Plant Nutrition and Fertilization in Low-Middle Reaches of the Yangtze River, Ministry of Agriculture, Nanjing Agricultural University, Nanjing, 210095, China

^5^ School of Life Sciences, Centre for Cell & Developmental Biology and State Key Laboratory of Agrobiotechnology, The Chinese University of Hong Kong, Shatin, Hong Kong, 999077, China.

^6^ Institute of Leisure Agriculture, Jiangsu Academy of Agricultural Sciences, Nanjing, 210014, China.

^#^ These authors contributed equally to this work.

^*^The corresponding author: Xueying Guan ([xueyingguan@zju.edu.cn](mailto:xueyingguan@zju.edu.cn))

**Supplemental materials**


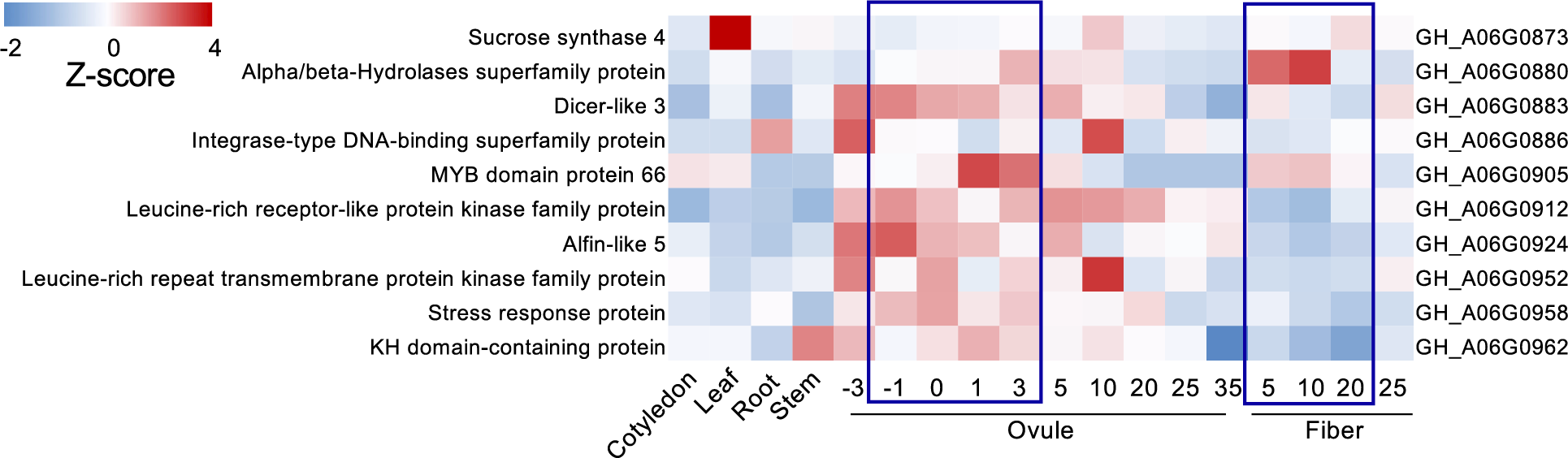


**Supplemental Figure 1**

Heatmap (Z-score) shows the tissue-specific expression pattern of the genes (RPKM > 1, in 1-DPA ovule) falling in the QTL-LP-A06 locus. The expression level of candidate genes at the stages of -1- to 3-DPA ovules and 5- to 20-DPA fibers were highlighted.


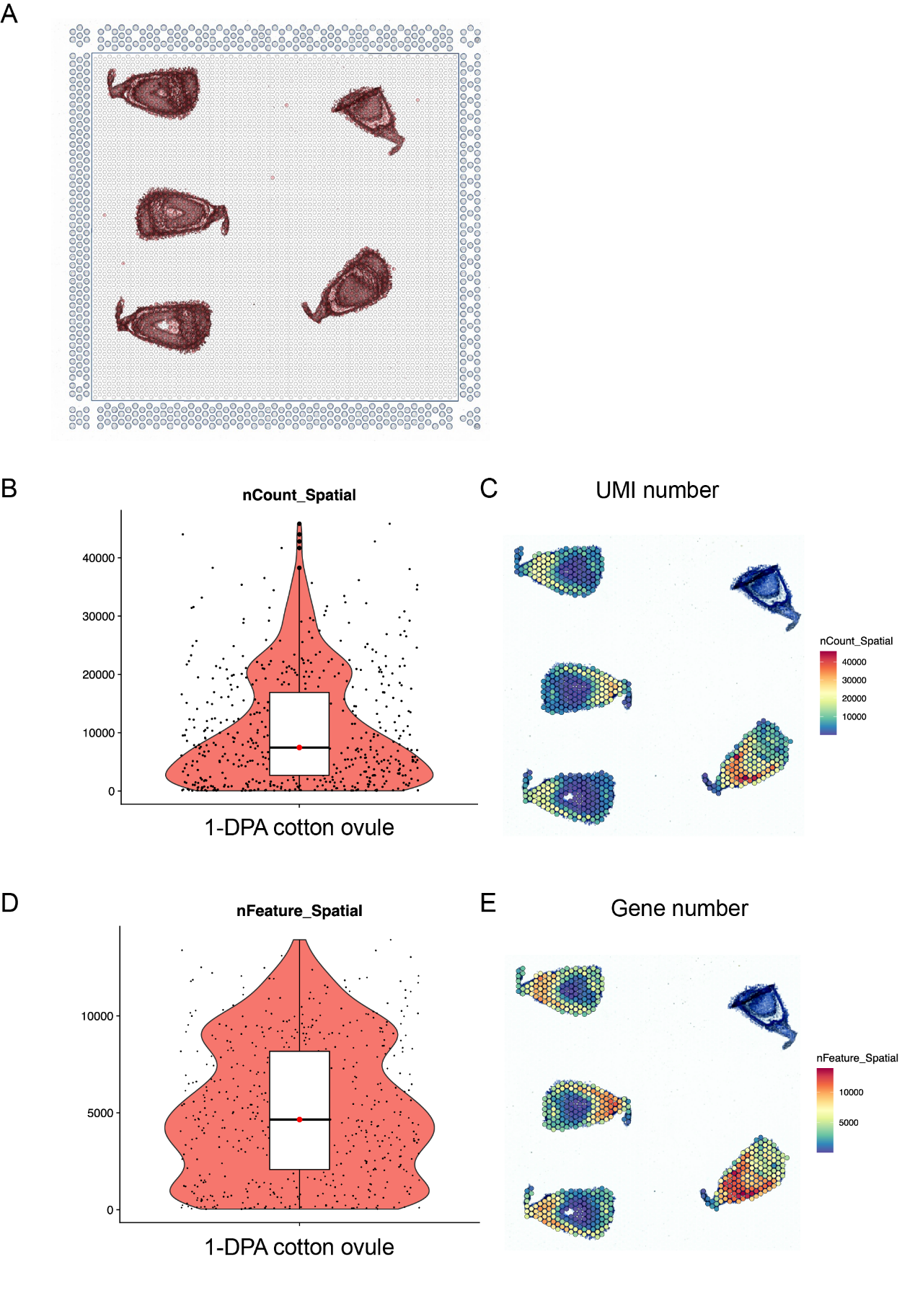


**Supplemental Figure 2: The** **spatial transcriptome data of 1-DPA cotton ovules.**

**A**: The 1-DPA ovule longitudinal sections for spatial transcriptome assays. **B**: The violin plot shows the unique molecular identifier (UMI) number for 1-DPA cotton ovules. **C**: The heatmap shows the UMI number distribution of the spatial transcriptome on 1-DPA ovule longitudinal sections. The upper right section of the ovule was excluded from the statistics because of the fold of tissues. **D**: The violin plot shows the gene number for 1-DPA cotton ovule. **E**: The heatmap shows the gene number distribution of the spatial RNA-seq on 1-DPA ovule longitudinal sections.


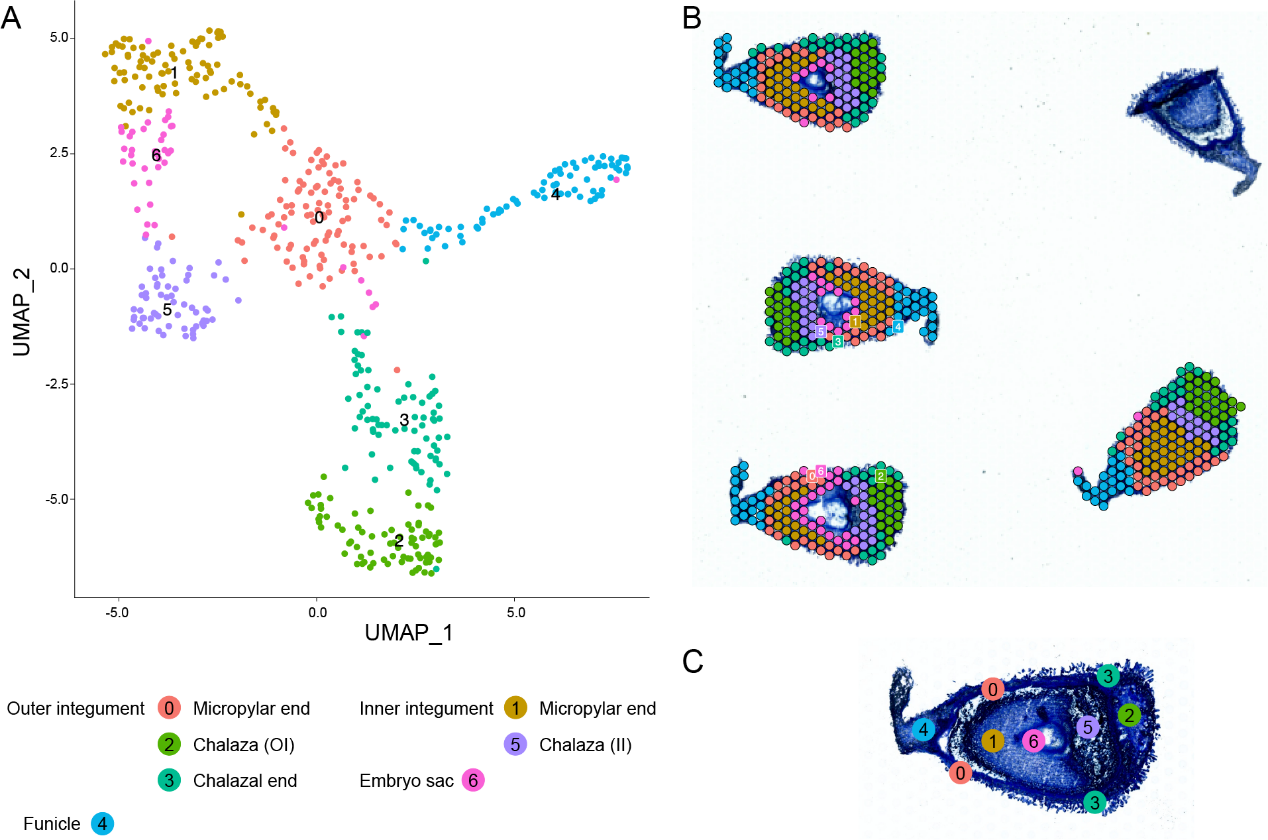


**Supplemental Figure 3: The 1-DPA Cotton Ovule Spatial Transcriptome Atlas (1-COSTA).**

**A**: UMAP plot representation of all cell types colored by seven clusters. **B**: Assemble maps that localize specific cell subtypes spatially within the 1-DPA ovule sections. **C**: The 1-DPA cotton ovule dissection was labeled with the barcode. Spatial barcoding data used to localize cell types in panels **B** and **C** were identical to those shown in the UMAP plot in panel **A**.


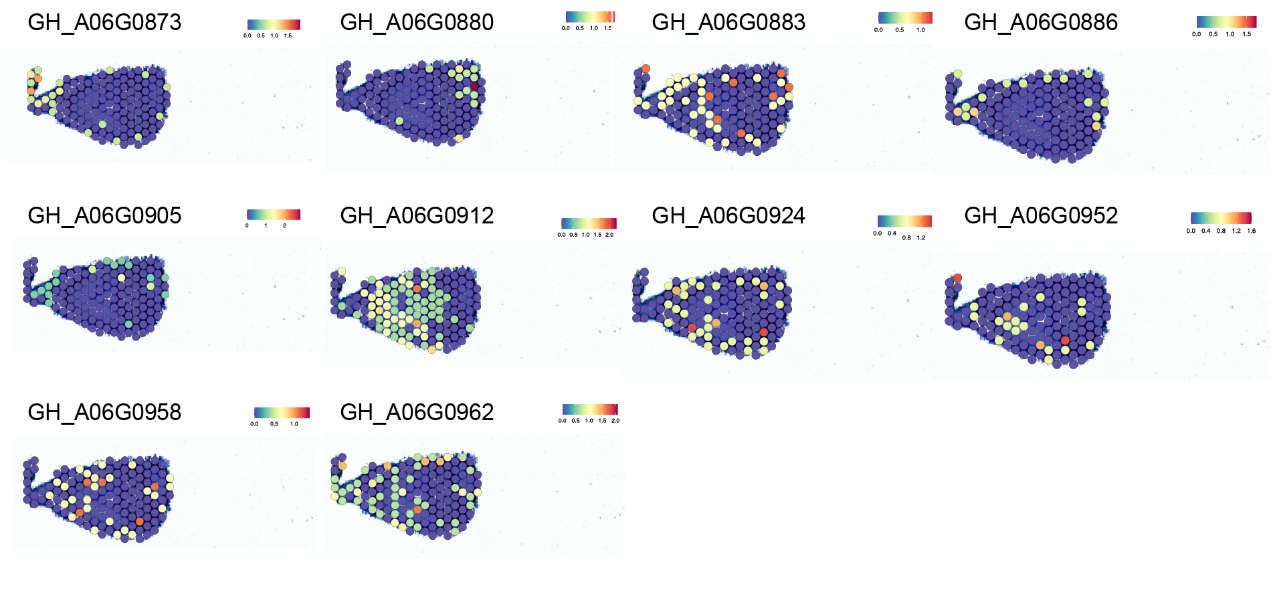


**Supplemental Figure 4**

**Heatmap from 1-COSTA to illustrate the spatial transcriptomic expression pattern of candidate genes in QTL-LP-A06 locus.**


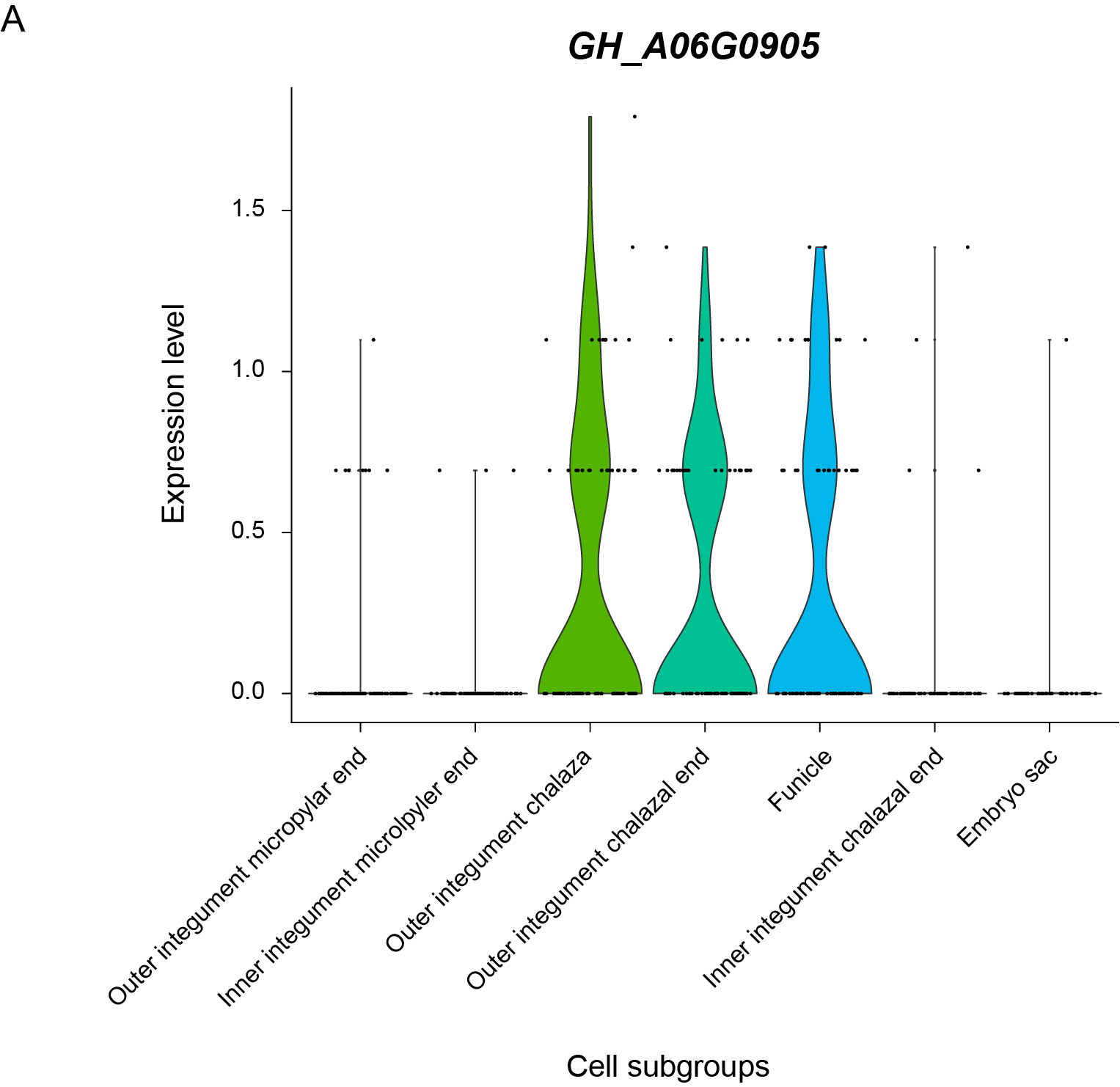


**Supplemental Figure 5**. **The spatial expression pattern of *GhLPF1* in 1-DPA ovule.**

A: The violin plot shows the expression level of *GhLPF1* in seven cell subgroups.


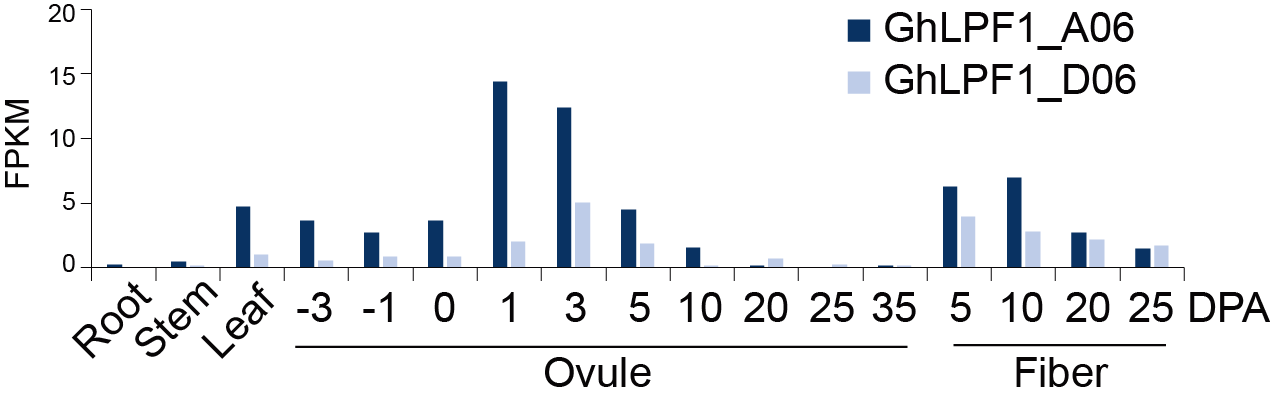


**Supplemental Figure 6. The Transcriptional level of *GhLPF1_A06* and *GhLPF1_D06* in cotton tissues.**


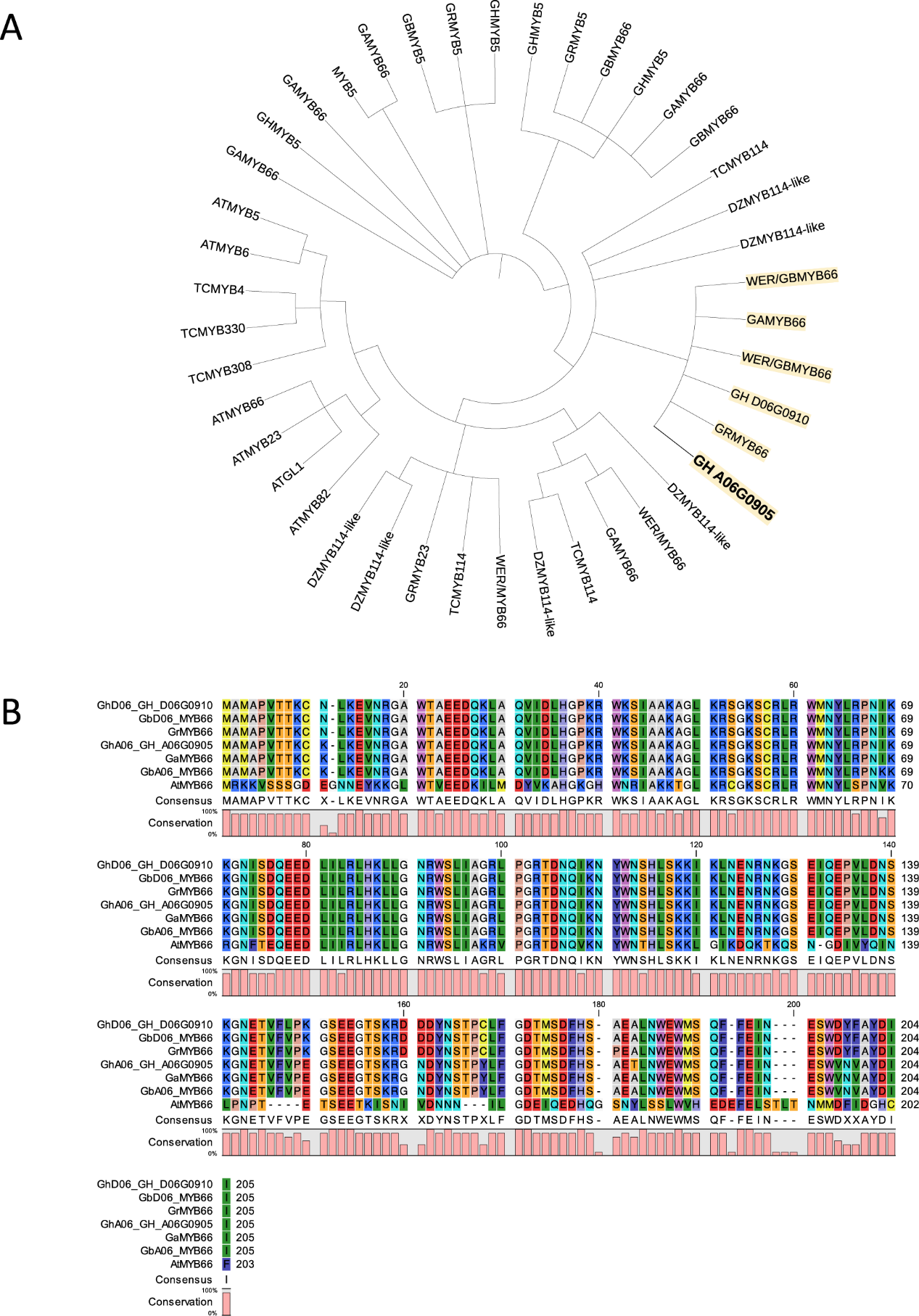


**Supplemental Figure 7: The *GhLPF1* is homologous to *AtMYB66*.**

**A**: The phylogenetic tree of MYB homologs on subgroup 15. **B**: The amino-acid sequence alignment of GhLPF1 in *Gossypium* lineage compared with AtMYB66.


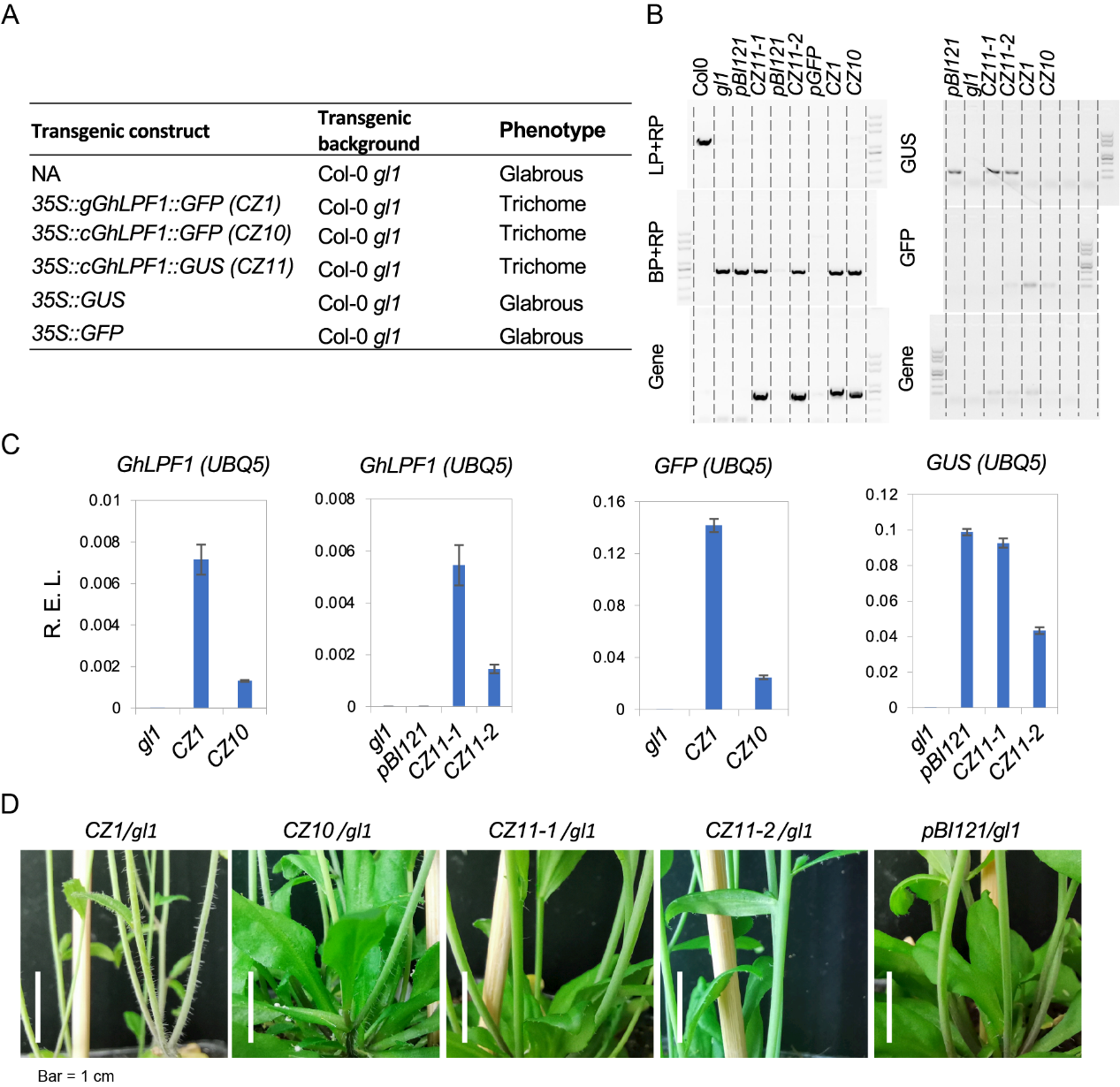


**Supplemental Figure 8: The functional test of *GhLPF1* on plant trichome regulation in *Arabidopsis***.

**A**: The table lists the constructs and the transgenic plant background information. **B**: The gel plot shows the genotyping results of the transgenic lines listed in Panel **A**. **C**: The histograms show the *GhLPF1* RNA relative expression level in the transgenic *Arabidopsis*. **D**: The photo images show the trichome growth on leaf and stem tissue in the corresponding transgenic lines.


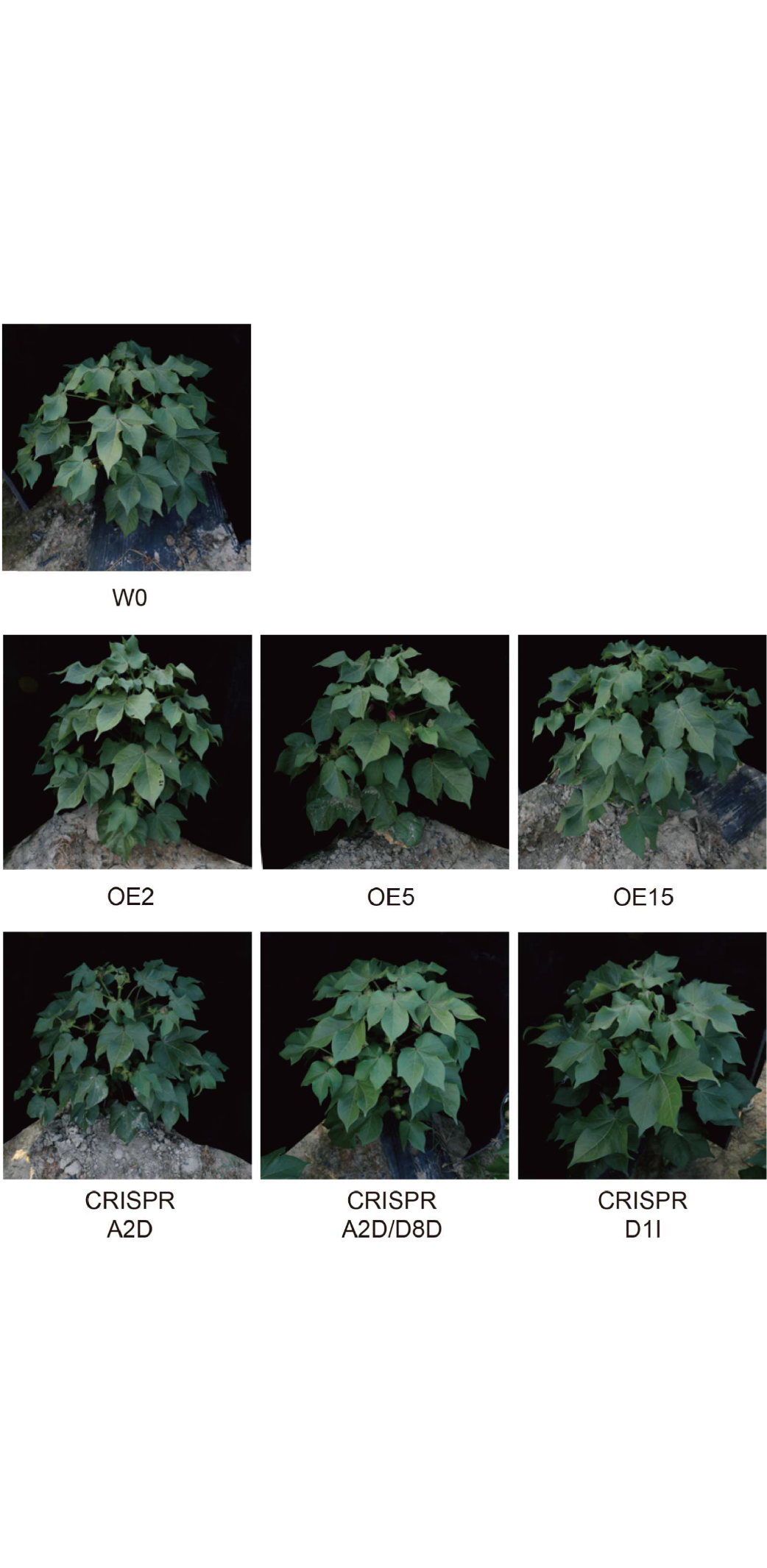


**Supplemental Figure 9: The photo images of mature cotton plants from the W0 and *GhLPF1* transgenic lines.**


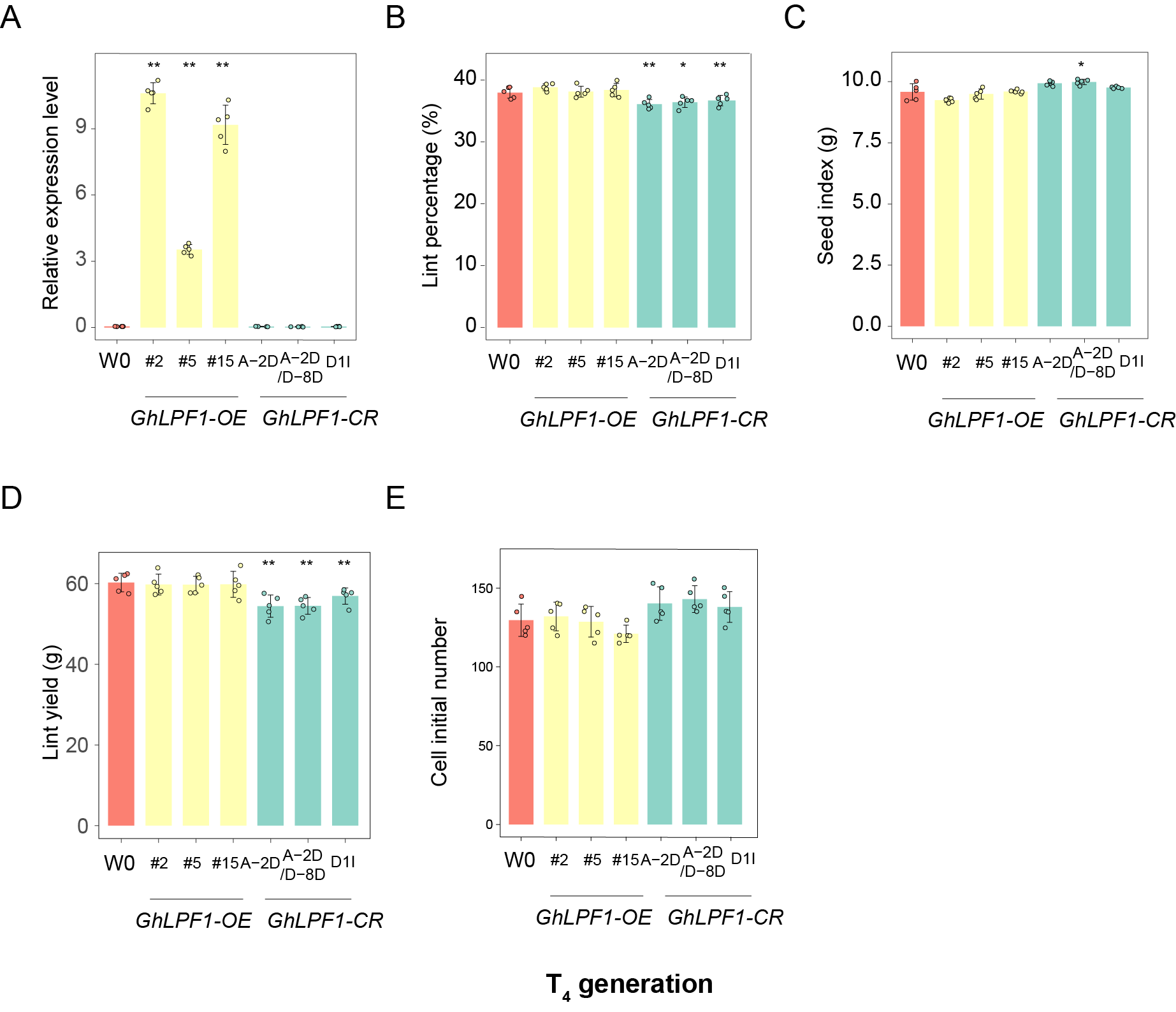


**Supplemental Figure 10: The phenotypic data of W0 and *GhLPF1* transgenic lines.**

The histograms show the relative expression level of *GhLPF1* (A), LP (B), SI (C), lint yield (D), and fiber cell initial number (E) of W0 and T_4_ generation *GhLPF1* transgenic lines. Values are means ± SD of five biological replicates (*n* = 5). The data were statistically analyzed by two-tailed Student’s *t*-test (**P* < 0.05, ***P* < 0.01). The phenotypic data were collected from the 2022.4-2022.10 farming season in Hangzhou, Zhejiang China.


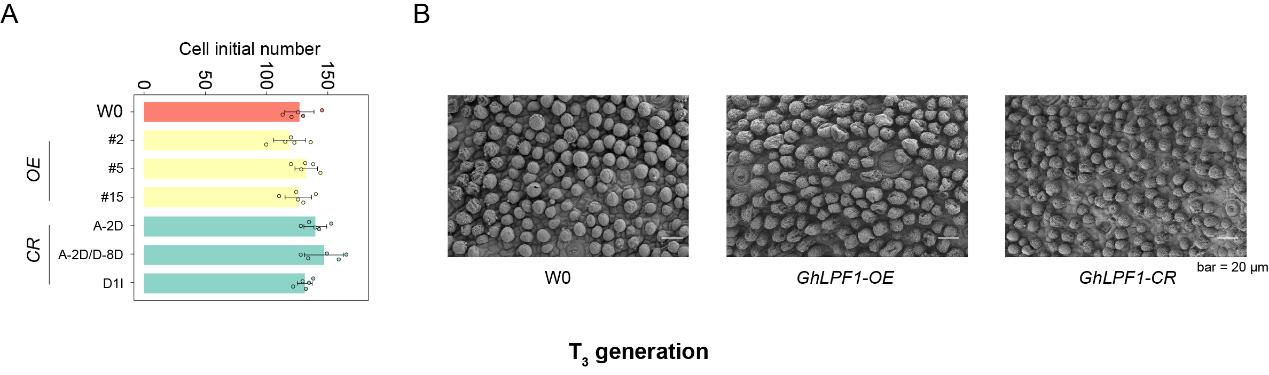


**Supplemental Figure 11: The fiber cell initial number of W0 and *GhLPF1* transgenic lines.**

(A): The fiber cell initial number of W0 and *GhLPF1* transgenic lines in T_3_ generation. Values are means ± SD of five biological replicates (*n* = 5). (B): The scanning electron microscope (SEM) photo images show the epidermis of 1-DPA ovules of W0 and *GhLPF1* transgenic lines.


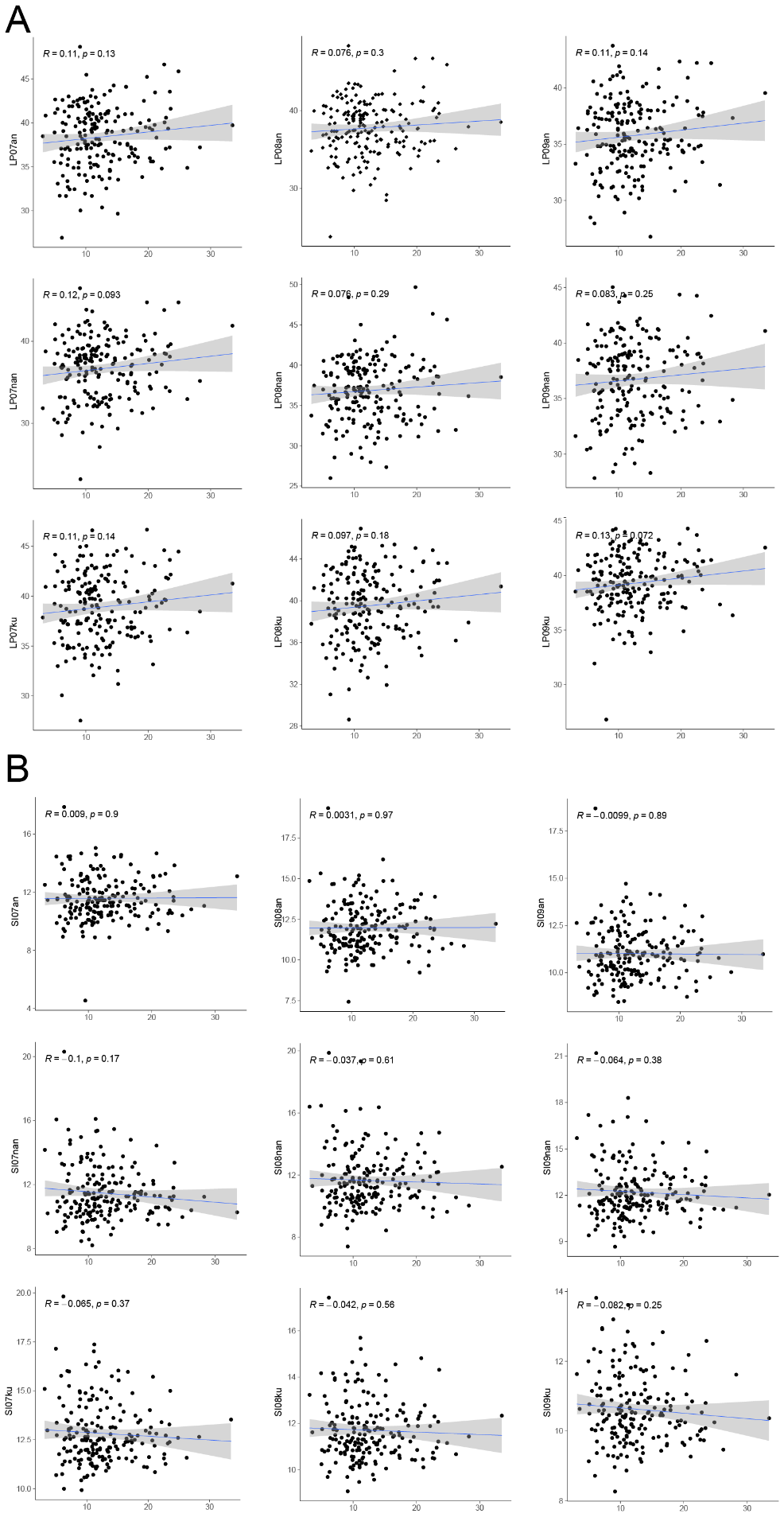


**Supplemental Fig 12: The linear regression analysis between *GhLPF1* mRNA level and LP or SI in cotton cultivated population.**

**A**: Linear regression analysis of *GhLPF1* expression level and LP in different farming years and locations. B: Linear regression analysis of *GhLPF1* expression level and SI in different farming years and locations. The population RNA-seq and traits data were adapted from the previous publication. Pearson correlations and *P*-value were calculated using the R function: lm (y∼x, data).


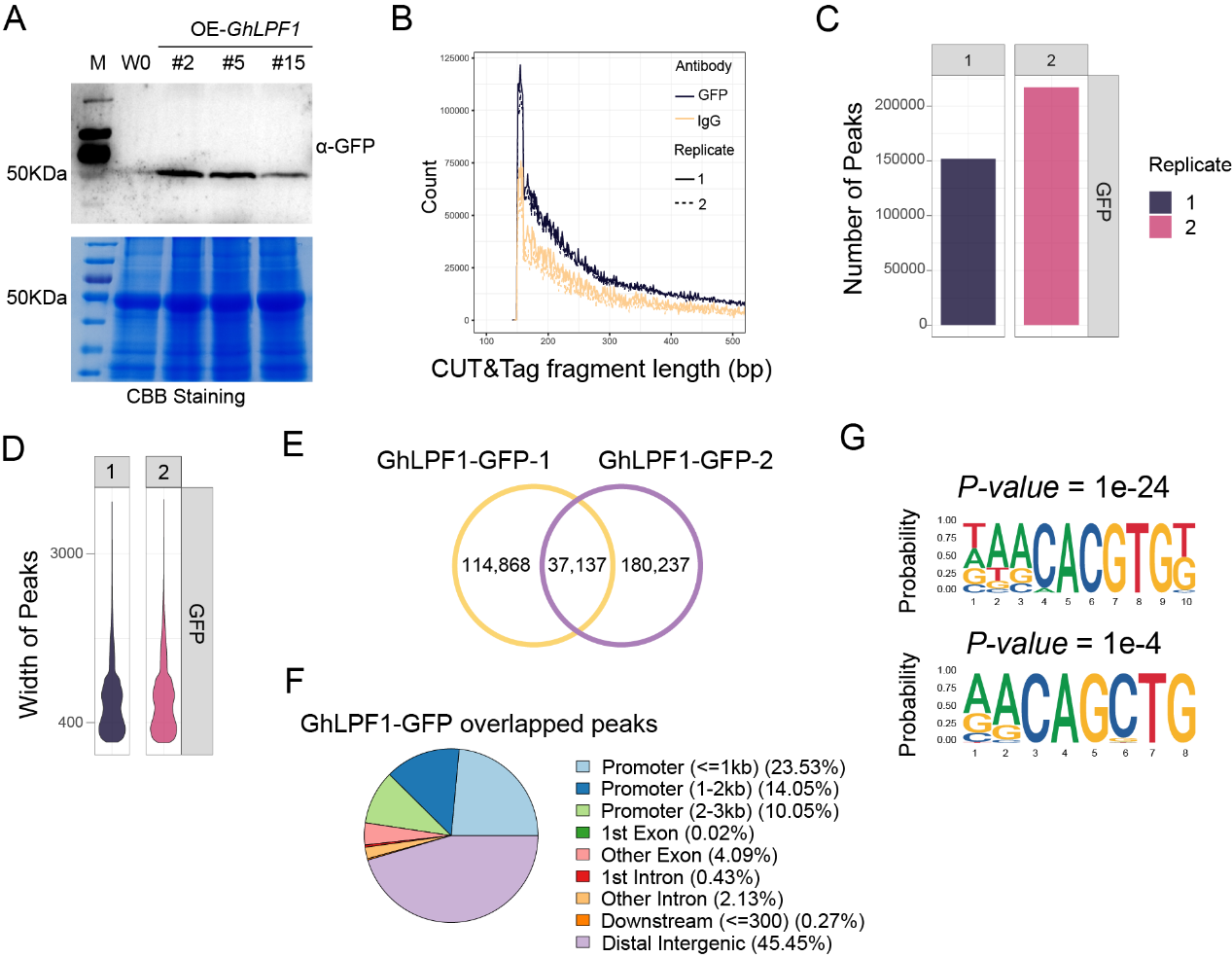


**Supplemental Figure 13: The quality control of CUT&Tag-Seq data for GhLPF1.**

**A**: The western blot and Coomassie blue staining show the GhLPF1-GFP protein in the *GhLPF1*-OE lines, while the W0 serves as a negative control. **B**: The line chart shows the distribution of fragment length in CUT&Tag-Seq. **C**: The bar plot shows the number of peaks called after mapping against the reference genome for two technical replicates. **D**: The violin plot shows the width of peaks in CUT&Tag-Seq. **E**: The Venn diagram shows the number of overlapped peaks from two technical replicates. **F**: The pie chart shows the genomic distribution of the overlapped peaks from two technical replicates. **G**: The enriched motif of GhLPF1 binding peaks.


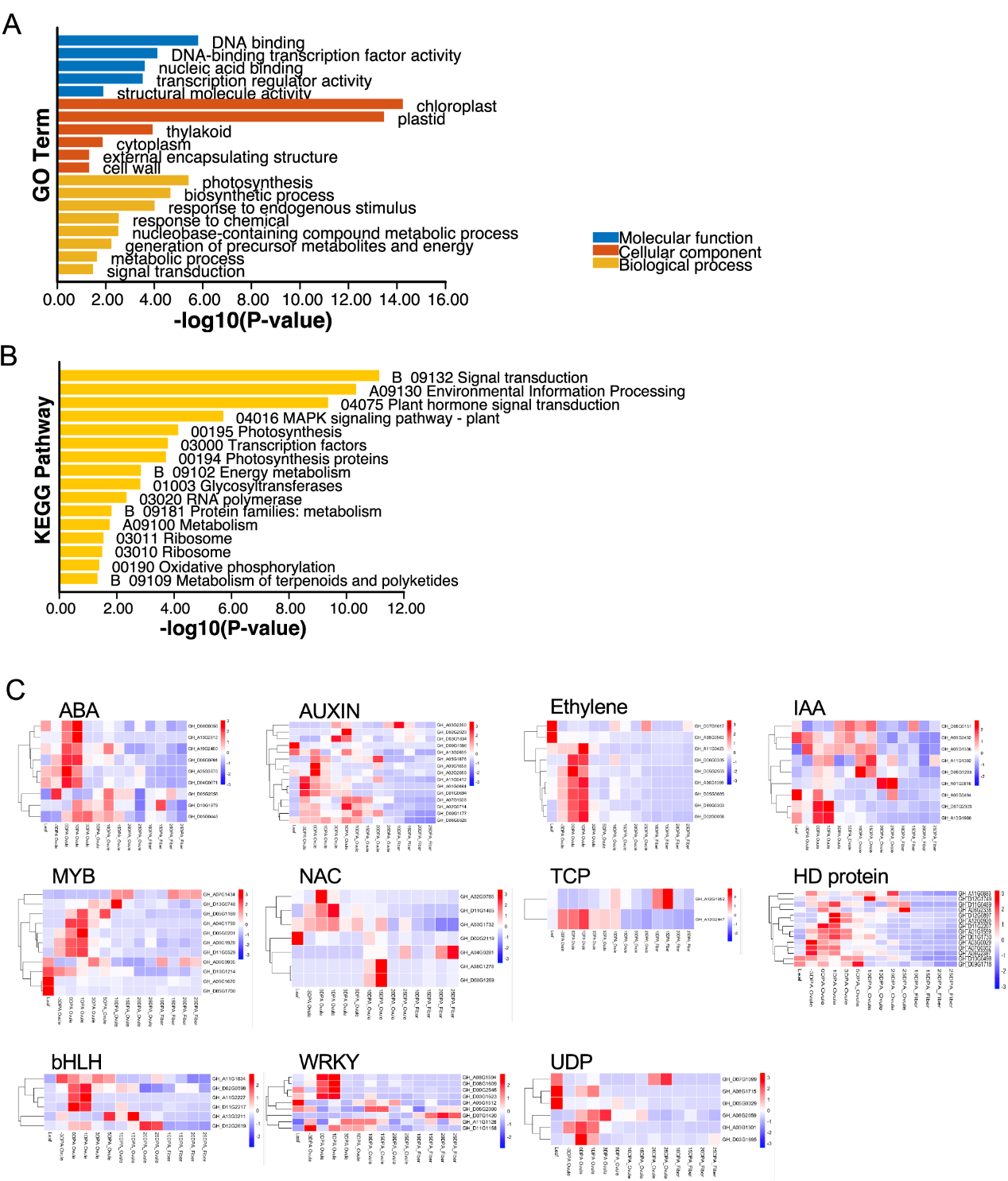


**Supplemental Figure 14: The functional enrichments of GhLPF1 downstream genes revealed by CUT&Tag-Seq.**

**A**: The GO enrichment for GhLPF1 downstream genes revealed by CUT&Tag-Seq. **B**: The KEGG enrichment for GhLPF1 downstream genes revealed by CUT&Tag-Seq. **C**: Expression pattern of GhLPF1 downstream genes classified by the functional annotation of phytohormones (ABA, auxin, ethylene, IAA), preliminary transcription factors (MYB, NAC, TCP, HD, bHLH, WRKY), and UDP. The cotton tissues included leaf, ovules (-3, 0, 1, 3, 5, 10, 15, 20, 25 DPA) and fibers (10, 15, 20, 25 DPA).


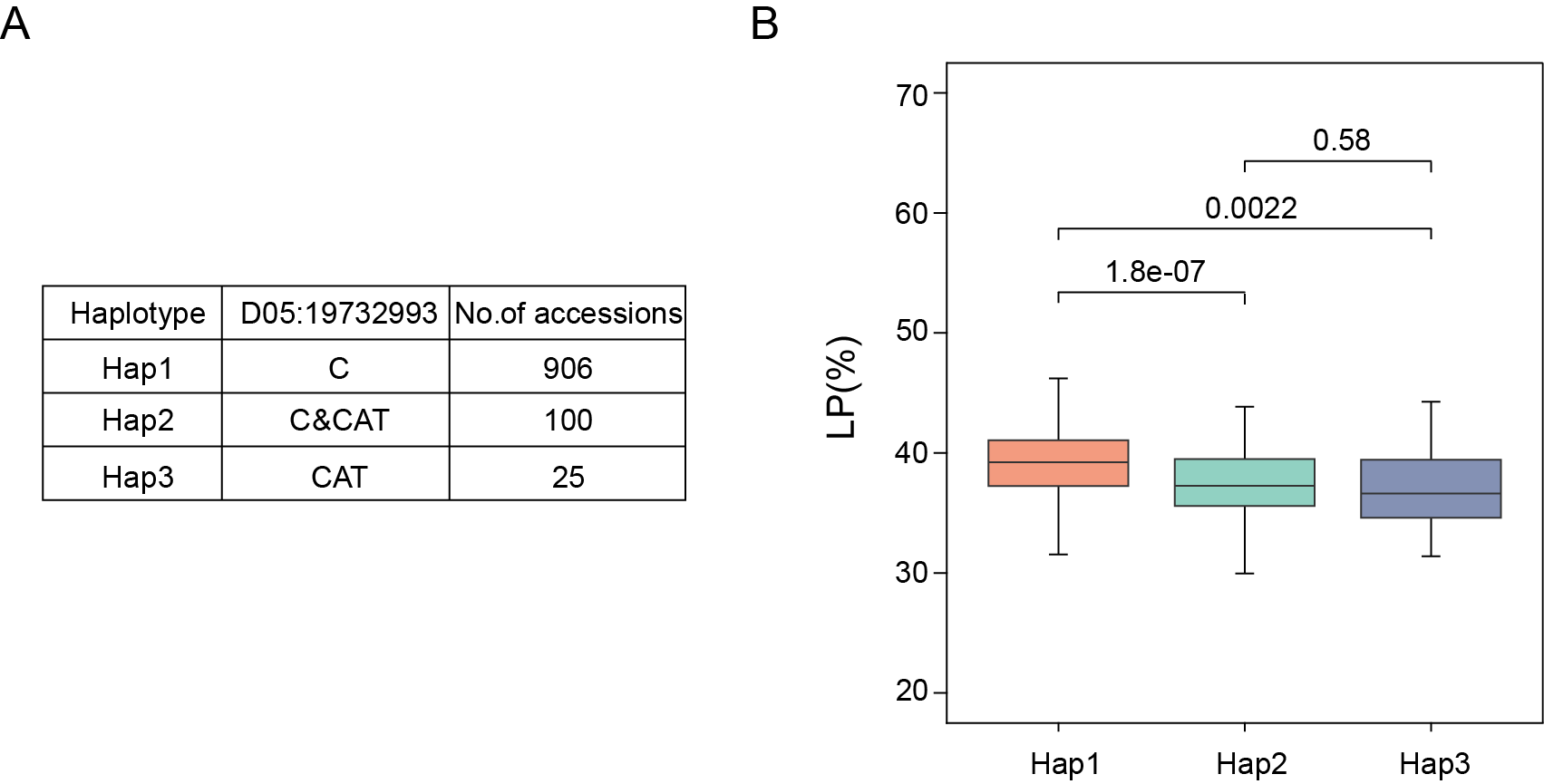


**Supplemental Figure 15: The genetic effect of *GhHB6* natural variation.**

**A:** The three haplotypes of *GhHB6* in upland cotton population.

**B:** The box plot shows the associated LP value of accessions with three *GhHB6* haplotypes. Pair comparisons were made by a two-tailed Student’s *t*-test.


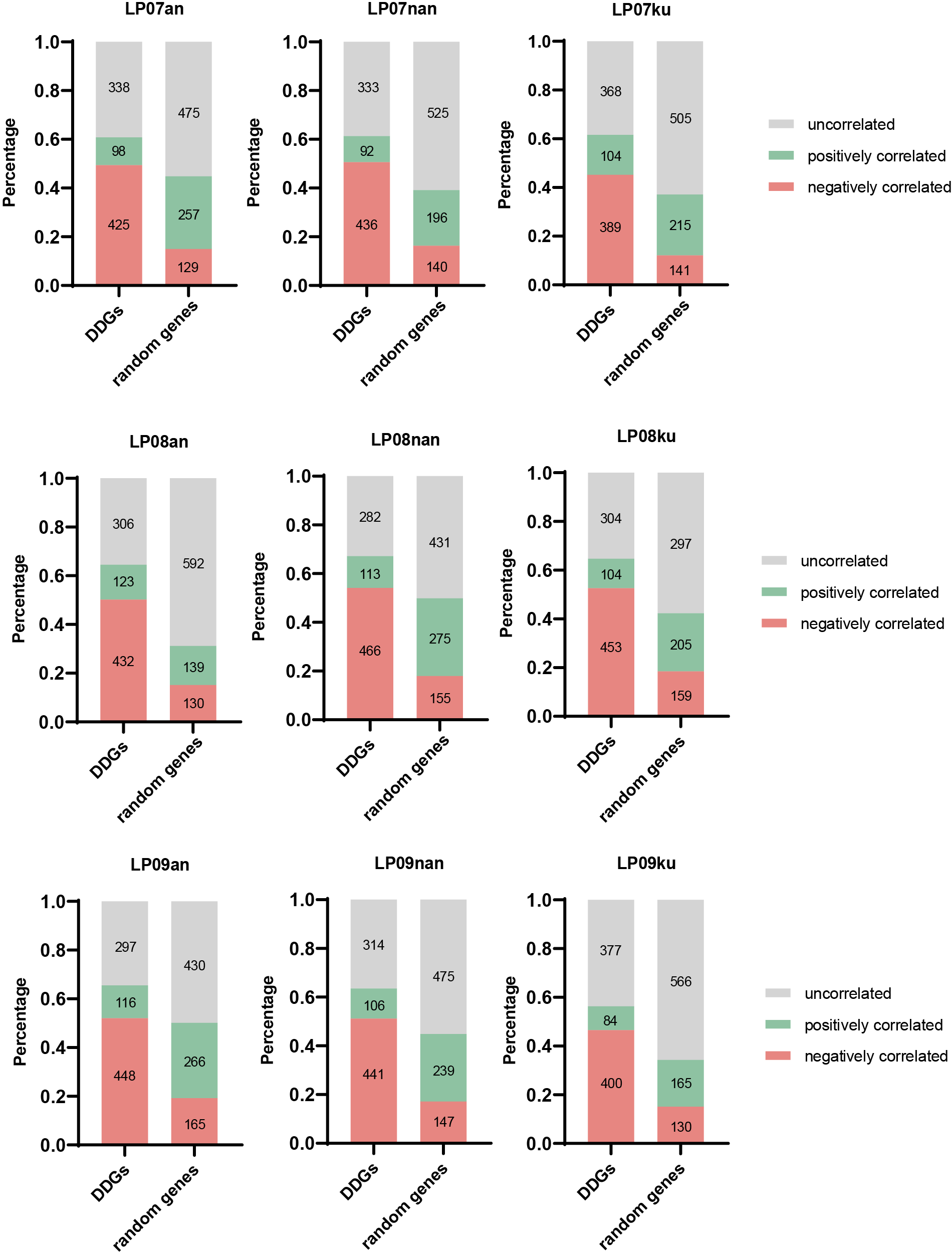


**Supplemental Figure 16: The correlation** **statistics of GhLPF1-DDGs expression level and LP in CUCP1.**

The bar plots show the percentage of three different correlation relationships regarding the expression level of 861 GhLPF1-DDGs and LP in CUCP1. The “uncorrelated” indicates the *P-*value of correlation exceeding the threshold of 0.05 (*P* > 0.05), the “positively correlated” refers to the *P-*value of correlation that is below the threshold of 0.05 and the correlation coefficient that is greater than 0 (*P* < 0.05, *R* > 0), and the “negatively correlated” refers to the *P-*value of correlation that is below 0.05 and the correlation coefficient that is below 0 (*P* < 0.05, *R* < 0). 07an, 07nan, 07ku, 08an, 08nan, 08ku, 09an, 09nan and 09ku refer to different farming years and locations.


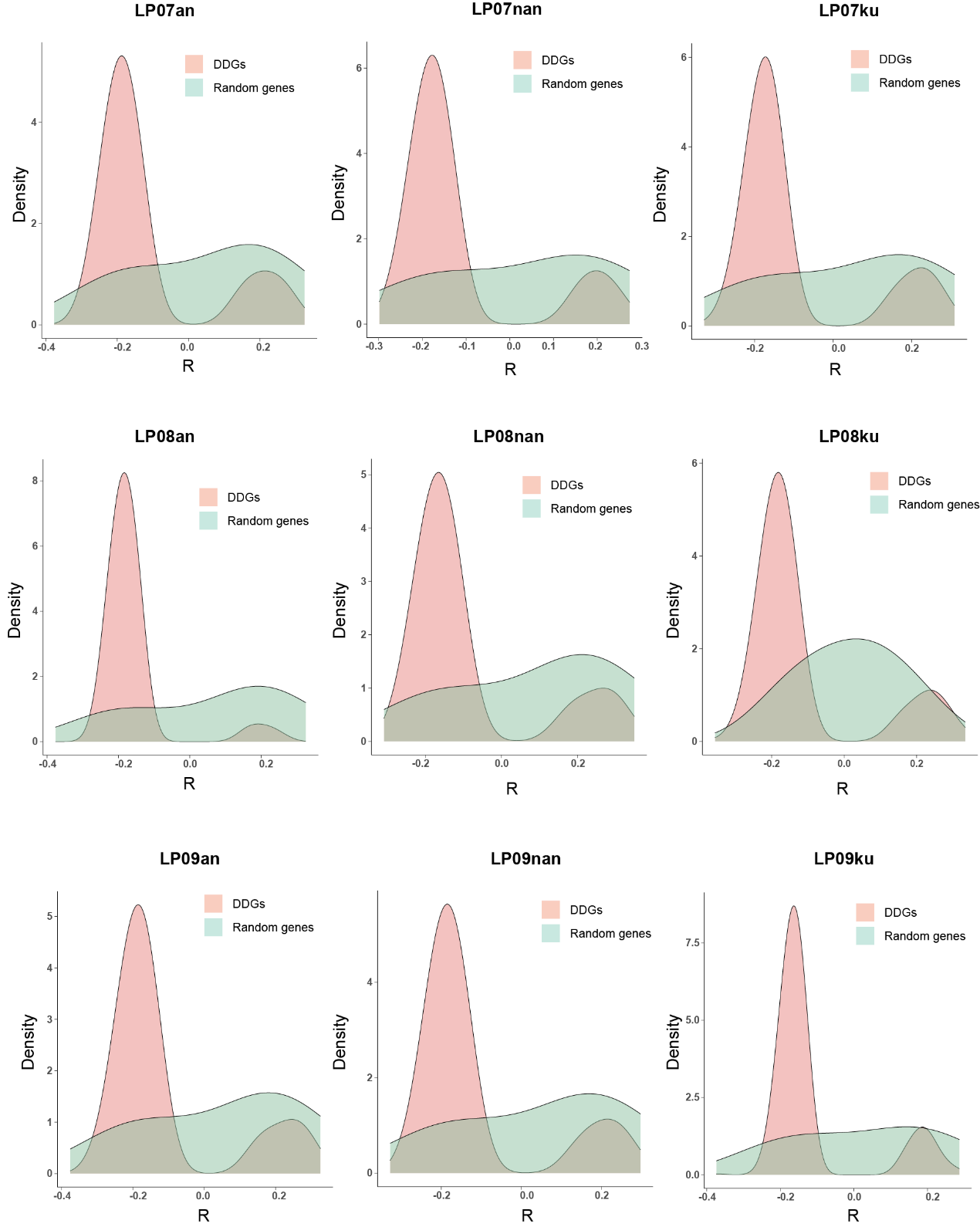


**Supplemental Figure 17: The correlation distribution of GhLPF1-DDGs expression level and LP in CUCP1.**

The density plots show the distribution of the correlation coefficient (*R*) of GhLPF1-DDGs expression level and LP is biased to negative numbers, while the random genes have no obvious distribution preference.


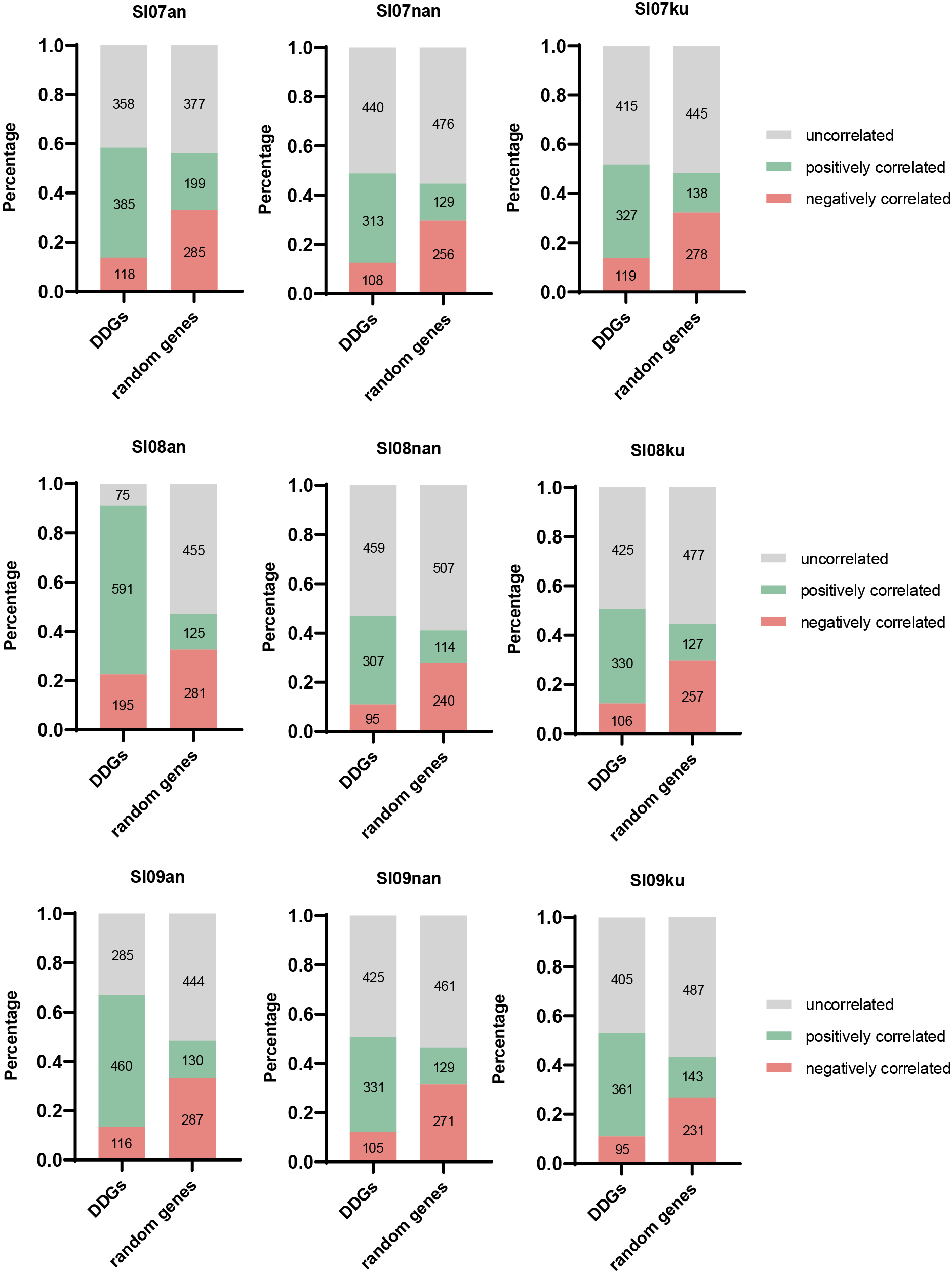


**Supplemental Figure 18: The correlation** **statistics of GhLPF1-DDGs expression level and SI in CUCP1.**

The bar plots show the percentage of three different correlation relationships of 861 GhLPF1-DDGs expression level and SI in CUCP1. The “uncorrelated” indicates the *P-*value of correlation exceeding the threshold of 0.05 (*P* > 0.05), the “positively correlated” refers to the *P-*value of correlation that is below the threshold of 0.05 and the correlation coefficient that is greater than 0 (*P* < 0.05, *R* > 0), and the “negatively correlated” refers to the *P-*value of correlation that is below 0.05 and the correlation coefficient that is below 0 (*P* < 0.05, *R* < 0).


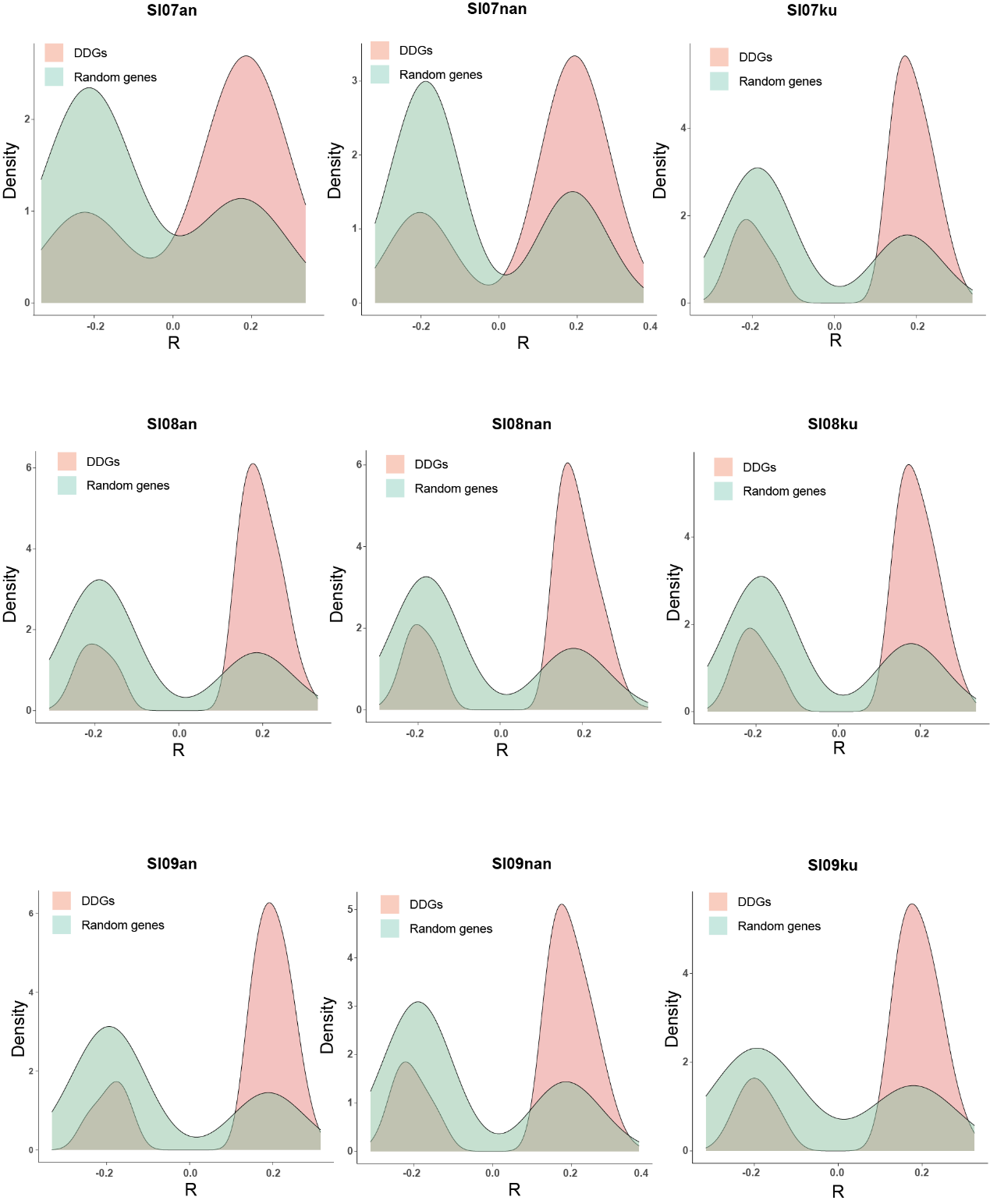


**Supplemental Figure 19: The correlation distribution of GhLPF1-DDGs expression level and SI in CUCP1.**

The density plots show that the distribution of the correlation coefficient (*R*) of GhLPF1-DDGs expression level and SI is biased to positive numbers, while the random genes have no obvious distribution preference.


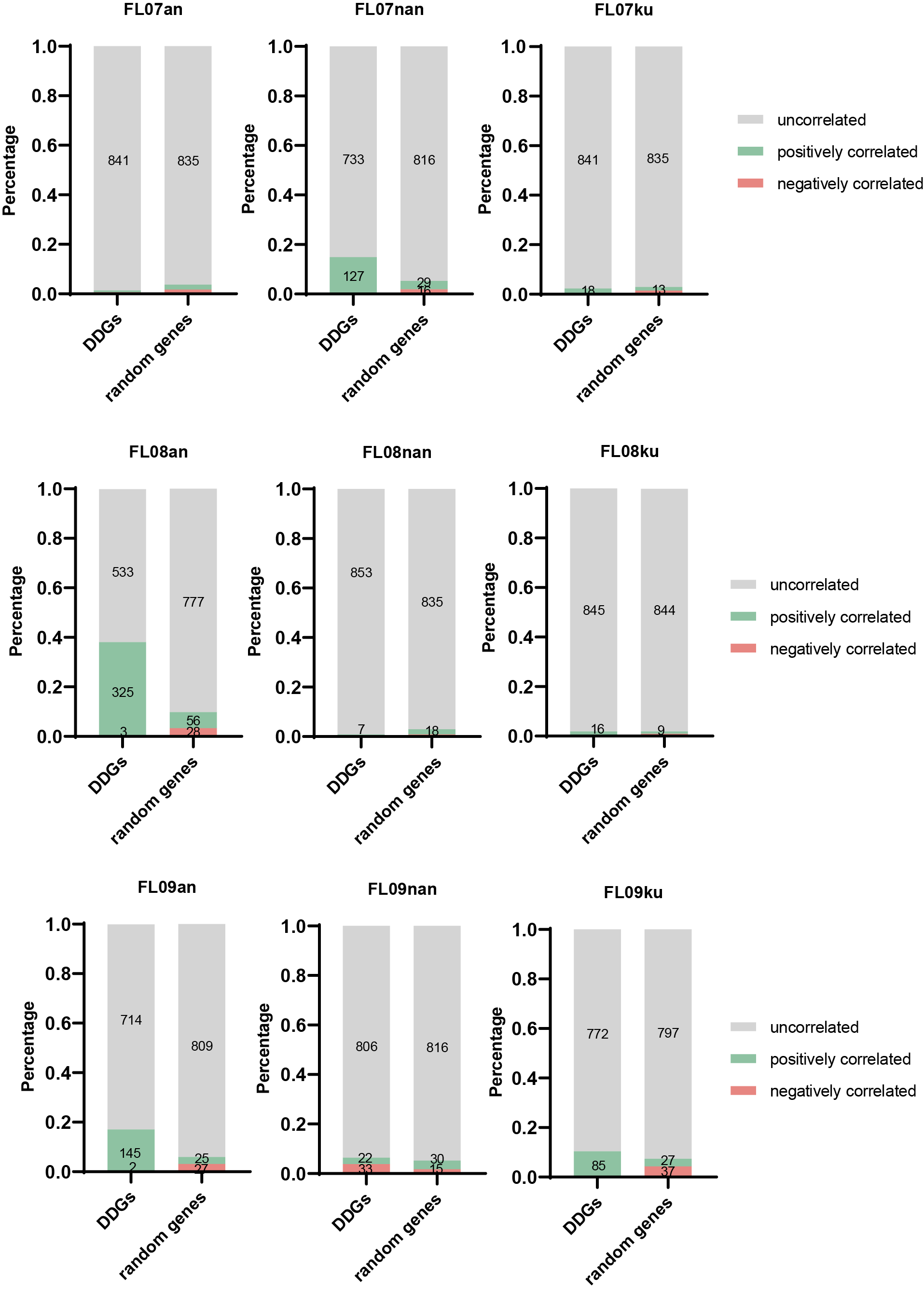


**Supplemental Figure 20: The correlation statistics of GhLPF1-DDGs expression level and FL in CUCP1.**

The bar plots show the percentage of three different correlation relationships of 861 GhLPF1-DDGs expression level and FL in CUCP1. The “uncorrelated” indicates the *P-*value of correlation exceeding the threshold of 0.05 (*P* > 0.05), the “positively correlated” refers to the *P-*value of correlation that is below the threshold of 0.05 and the correlation coefficient that is greater than 0 (*P* < 0.05, *R* > 0), and the “negatively correlated” refers to the *P-*value of correlation that is below 0.05 and the correlation coefficient that is below 0 (*P* < 0.05, *R* < 0).

**Supplemental Table list:**

Supplemental Table 1 initial QC for ST of 1-DPA cotton ovule

Supplemental Table 2 The number of spots in different cell clusters of ST at 1-DPA cotton ovule

Supplemental Table 3 The quality control data for RNA-seq

Supplemental Table 4 DEGs of RNA-seq

Supplemental Table 5 Summary of CUT&Tag-seq data

Supplemental Table 6 The summary for peak calling from CUT&Tag

Supplemental Table 7 Overlap genes of CUT&Tag-Seq and RNA-Seq

Supplemental Table 8 Transcription factors in the overlap genes of CUT&Tag and RNA-Seq

Supplemental Table 9 The primers used in this study
